# Bioinformatics and machine learning were used to validate glutamine metabolism-related genes and immunotherapy in osteoporosis patients

**DOI:** 10.1186/s13018-023-04152-2

**Published:** 2023-09-14

**Authors:** Lei Wang, Chaosheng Deng, Zixuan Wu, Kaidong Zhu, Zhenguo Yang

**Affiliations:** 1 Shandong University of Traditional Chinese Medicine, Jinan, China; 2 Second Affiliated Hospital of Shandong University of Traditional Chinese Medicine, Jinan, China; 3 The Third People Hospital of Longgang District, Shenzhen, Guangdong Province China; 4 Guangzhou University of Chinese Medicine, Guangzhou, Guangdong Province China

**Keywords:** Osteoporosis (OP), Gln-metabolism genes (GlnMgs), DEGs, WGCNA, Bioinformatics

## Abstract

**Background:**

Osteoporosis (OP), often referred to as the “silent disease of the twenty-first century,” poses a significant public health concern due to its severity, chronic nature, and progressive course, predominantly affecting postmenopausal women and elderly individuals. The pathogenesis and progression of this disease have been associated with dysregulation in tumor metabolic pathways. Notably, the metabolic utilization of glutamine has emerged as a critical player in cancer biology. While metabolic reprogramming has been extensively studied in various malignancies and linked to clinical outcomes, its comprehensive investigation within the context of OP remains lacking.

**Methods:**

This study aimed to identify and validate potential glutamine metabolism genes (GlnMgs) associated with OP through comprehensive bioinformatics analysis. The identification of GlnMgs was achieved by integrating the weighted gene co-expression network analysis and a set of 28 candidate GlnMgs. Subsequently, the putative biological functions and pathways associated with GlnMgs were elucidated using gene set variation analysis. The LASSO method was employed to identify key hub genes, and the diagnostic efficacy of five selected GlnMgs in OP detection was assessed. Additionally, the relationship between hub GlnMgs and clinical characteristics was investigated. Finally, the expression levels of the five GlnMgs were validated using independent datasets (GSE2208, GSE7158, GSE56815, and GSE35956).

**Results:**

Five GlnMgs, namely IGKC, TMEM187, RPS11, IGLL3P, and GOLGA8N, were identified in this study. To gain insights into their biological functions, particular emphasis was placed on synaptic transmission GABAergic, inward rectifier potassium channel activity, and the cytoplasmic side of the lysosomal membrane. Furthermore, the diagnostic potential of these five GlnMgs in distinguishing individuals with OP yielded promising results, indicating their efficacy as discriminative markers for OP.

**Conclusions:**

This study discovered five GlnMgs that are linked to OP. They shed light on potential new biomarkers for OP and tracking its progression.

**Supplementary Information:**

The online version contains supplementary material available at 10.1186/s13018-023-04152-2.

## Introduction

In 1993, the World Health Organization (WHO) officially classified osteoporosis as a systemic skeletal disease characterized by reduced bone mass, microarchitectural deterioration of bone tissue, heightened bone fragility, and increased susceptibility to fractures [1]. This condition has earned the alarming moniker “the silent epidemic of the twenty-first century” due to its profound impact on public health [2]. Osteoporosis represents the most prevalent among metabolic bone disorders, manifesting as a severe, chronic, and progressive ailment with subtle clinical manifestations [3]. Ranked as the fourth most prevalent chronic illness, after heart disease, dementia, and lung cancer, osteoporosis imposes substantial economic and social burdens worldwide, demanding urgent attention as a global public health concern [4]. Its asymptomatic nature until the occurrence of the first osteoporotic fracture often leads to misdiagnoses, underscoring the pressing need to identify early diagnostic biomarkers [5].

All living creatures require the ability to absorb nutrition and undeartake metabolism. Cancer has a feature called metabolic reprogramming, which promotes tumor cell multiplication and survival [6]. Oncogenic transformation creates a well-defined metabolic phenotype in tumor cells, which modifies the tumor environment (TME), according to recent studies. TME is composed of multiple cell types in a complex matrix that is characterized by inefficient oxygen and nutrition delivery due to inadequate or poorly differentiated vasculature [7]. The study of non-tumor immune infiltration has steadily gained importance as research has progressed. More and more evidence shows that the immune response is associated with significant changes in tissue metabolism, such as nutritional depletion, increased oxygen use, and the generation of reactive nitrogen and oxygen intermediates [8]. Similarly, various microenvironmental substances impact immune cell development and function, implying that metabolic interventions may enhance the efficiency of immunotherapies [9].

Glutamine (Gln), as the most prevalent amino acid in circulation, exhibits rapid uptake in cultured tumor cells. Its role in cellular aerobic glycolysis, supporting TCA flow and serving as a citrate source in reductive carboxylation for lipid synthesis, has been widely studied. Additionally, glutaminolysis plays a crucial role in promoting proliferative cell survival by mitigating oxidative stress and preserving mitochondrial membrane integrity [10]. Notably, Gln utilization differs between M2 and naïve macrophages, influencing their inflammatory phenotypes, where reduced Gln metabolism favors proinflammatory M1 macrophages [11]. Manipulating Gln metabolism may offer a promising avenue to shift tumor-associated macrophages from M2 to M1, thereby enhancing the anti-tumor inflammatory immune response. Moreover, Gln metabolism has implications in Th1 cell differentiation and effector T cell activation, further suggesting its potential in reshaping the TME and improving immunotherapy efficacy. In the context of Alzheimer's disease, inflammasomes, large multiprotein complexes formed by specific pattern recognition receptors, play a critical role. Upon activation, inflammasomes induce the formation of membrane pores and process proinflammatory cytokines, culminating in pyroptosis, an inflammatory cell death pathway [12]. While targeting Gln in combination with immunotherapy holds great promise in the realm of oncology, the specific landscape of Gln metabolism in the context of Immunogenicity and Immunotherapy remains poorly understood. Given these knowledge gaps, our research aims to comprehensively evaluate the interplay of Gln metabolism and immunotherapy in the context of OP. Through this investigation, we seek to elucidate the potential of Gln-targeted approaches in optimizing immunotherapy strategies for OP treatment.

**Fig. 1:**
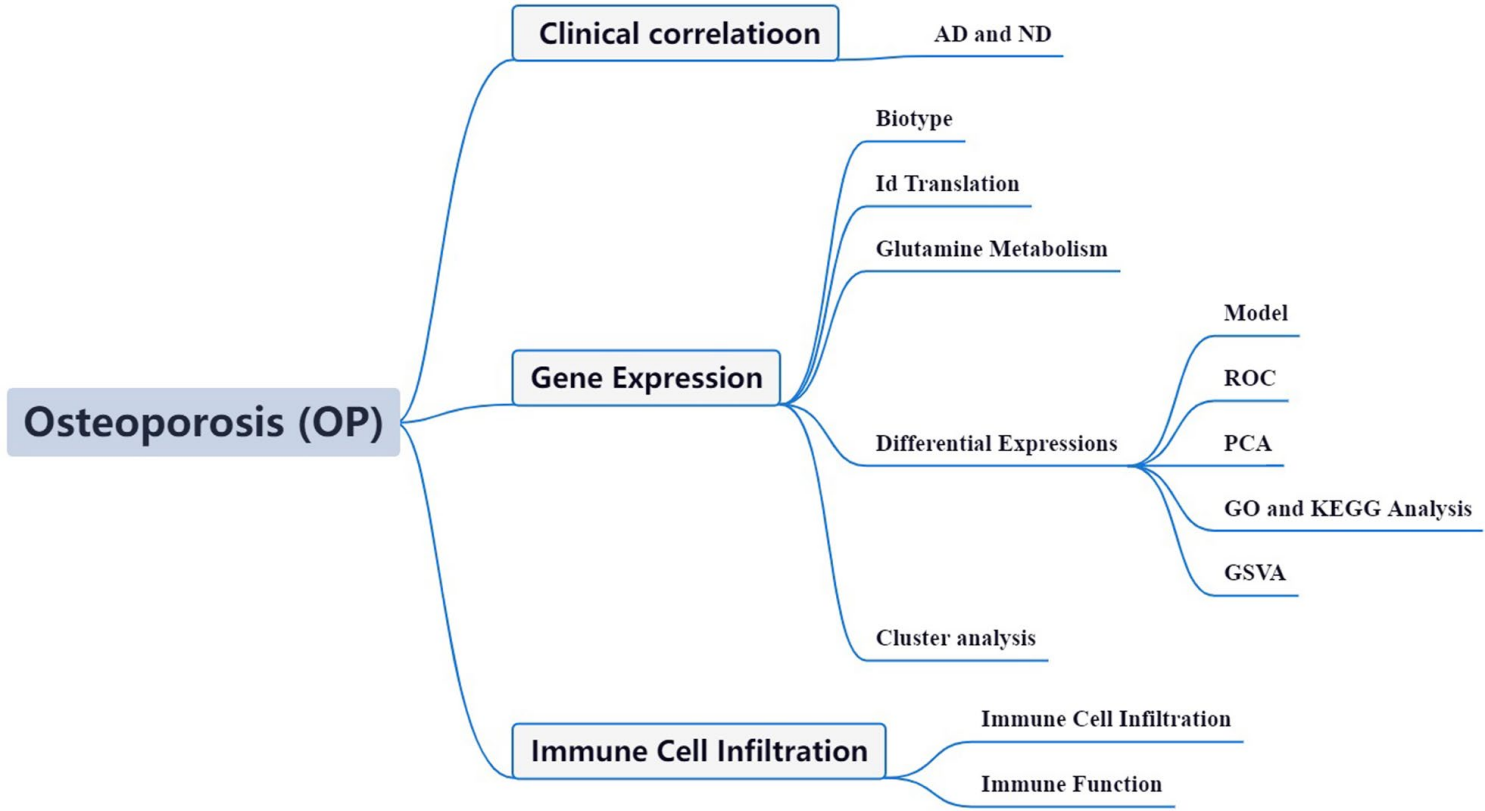
Framework

The OP Initiative's high-throughput transcriptome sequencing data and clinical annotation make it possible for us to examine the altered transcriptional and associated molecular pathways implicated in OP in biological research [13, 14]. The findings of these bioinformatics studies offer fascinating insights into the pathophysiology and mechanisms of OP from a variety of angles. However, no study has employed bioinformatics to assess if GlnMgs play a role in OP. As a result, the purpose of this study was to look at the OP-related GEO via the lens of the GlnMgs (Fig. 1).

## Materials and methods

The methods proposed by Zi-Xuan Wu et al. in 2023 were adopted in this study [15].

### Raw data

The GEO datasets GSE2208, GSE7158, GSE56815, and GSE35956, along with platforms GPL96 and GPL570, were utilized in the analysis. GSE2208 and GSE7158, GSE56815 were employed as the training dataset, while GSE35956 served as the test group. The MSigDB provided 79 GlnMgs (Additional file 1: Table S1).

### Analysis of differentially expressed genes (DEGs)

Precise mRNA data were obtained through Perl matching and sorting of transcription data. Following data standardization of GSE2208, GSE7158, and GSE56815, DEGs were identified using criteria of FDR < 0.05 and |log2FC|≥ 1 to assess changes in GlnMgs.

### Immune cell infiltration and cluster analysis

CIBERSORT was employed to analyze immune cell components. Prognosis-related GlnMgs were subjected to cluster analysis, resulting in the formation of two clusters, cluster 1 and cluster 2.

### Enrichment analysis

Biological functions and pathways were explored using Gene Ontology (GO) and Kyoto Encyclopedia of Genes and Genomes (KEGG). R was utilized to investigate the impact of differentially expressed GlnMgs on biological processes (BP), molecular functions (MF), and cellular components (CC). GSVA was used to compute the process.

### Co-expression gene identification

The WGCNA algorithm was used to categorize genes and identify relationships between modules and OP characteristics. The co-expression network was constructed using the top 25% variance genes from GSE2208, GSE7158, and GSE56815. The dynamic cutting tree approach with a threshold of 0.25 was employed to merge modules.

### GlnMgs identification

The identification of GlnMgs involved intersecting the DEGs from WGCNA, Gln, and cluster hub genes. The overlapping genes were visualized using Vnnmap, and their biological mechanisms and enrichment pathways were investigated as previously described. Hub GlnMgs were identified, and the GSE2208, GSE7158, and GSE56815 datasets were divided into training cohorts. External validation was conducted using the GSE35956 dataset. Finally, the prognosis of the test group was calculated by matching samples with age based on clinical information, and their correlation with age was explored.

### Drug-gene interactions

In the context of diagnosing illnesses, the development of biological models and identification of effective biomarkers have gained importance with the advancement of bioinformatics. However, the application of these biomarkers in clinical settings remains critical. Medication prediction based on useful markers becomes essential for future prevention and treatment of OP. The DGIdb database was utilized to predict drug interactions for the intersection gene in the XGB model and the generated hub genes, facilitating accurate medication predictions and serving as a reference for therapeutic interventions.

## Results

### DEG identification and principal component analysis

**Fig. 2:**
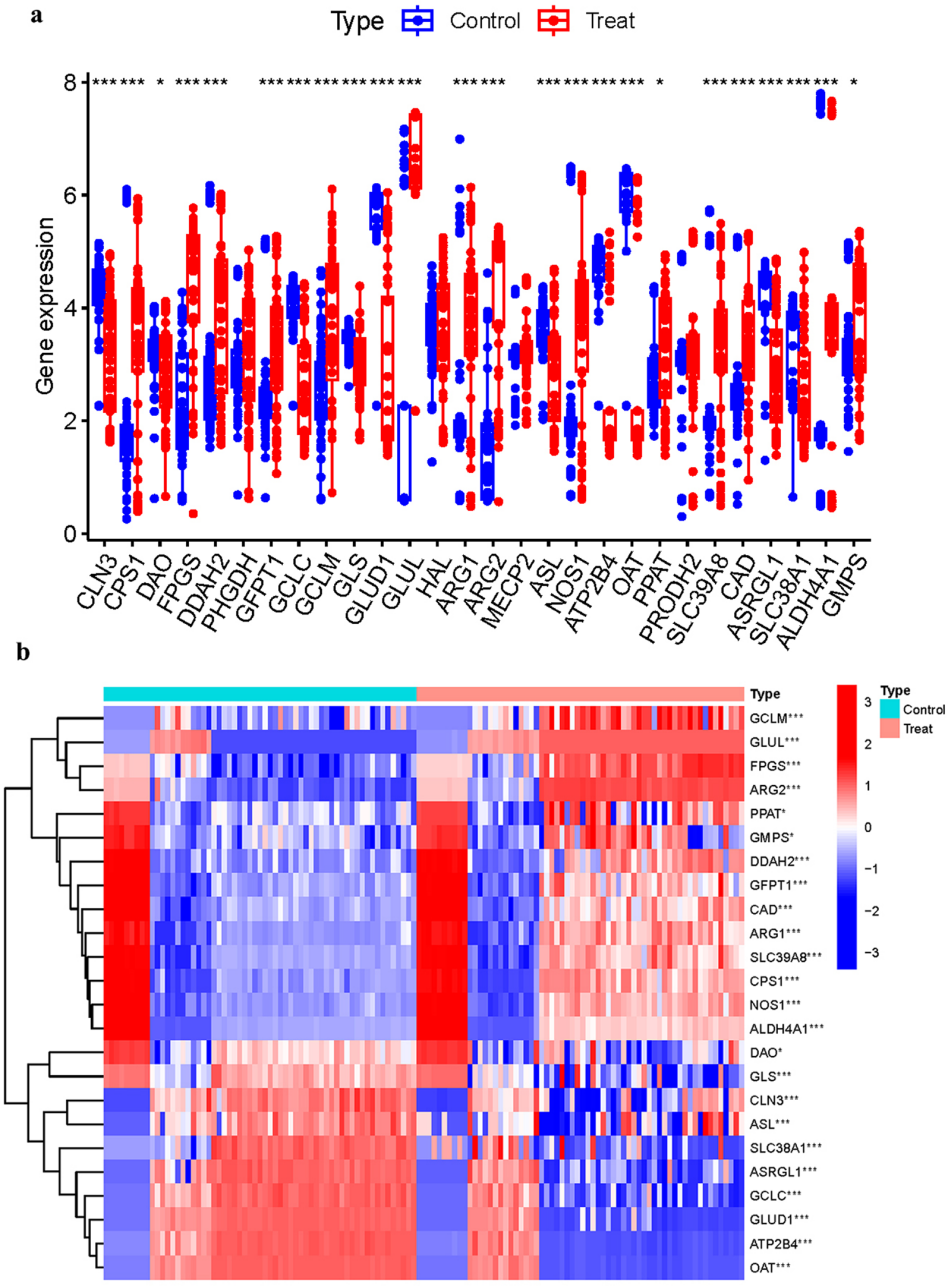
Principal Component Analysis. **a** 21 genes (CLN3, CPS1, FPGS, DDAH2, etc.) were found to be significantly different. **b** Some genes cluster in the treat group and some in the control group

Among the 24 GlnMgs, 21 genes (CLN3, CPS1, FPGS, DDAH2, etc.) were found to be significantly different (Fig. 2a). In addition, Some genes cluster in the treat group and some in the control group. Treat: GCLM, GLUL, FPGS, ARG2, CLN3, and ASL. Control: PPAT, GMPS, DDAH2, GFPT1, CAD, ARG1, SLC39A8, CPS1, NOS1, ALDH4A1, DAO, GLS, SLC38A1, ASRGL1, GCLC, GLUD1, ATP2B4, and OAT (Fig. 2b) (Additional file 1: Table S2).

### Expression of GlnMgs

**Fig. 3:**
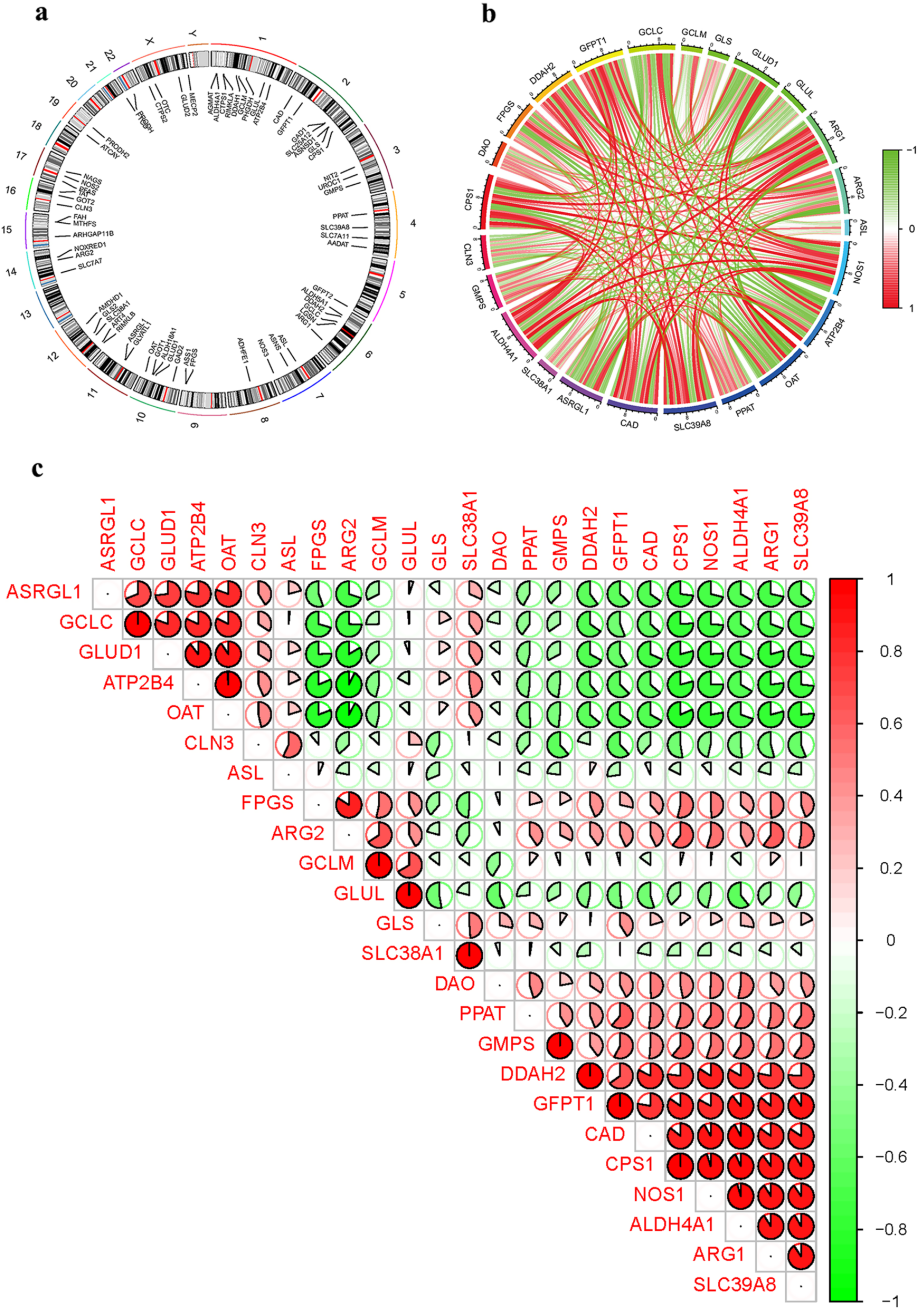
GlnMgs. **a** GlnMgs on sequences. **b**–**c** GlnMgs and related genes

GlnMgs chromosomal positions were calculated and visualized in circles (Fig. 3a) (Additional file 1: Table S3). Then, we conducted correlation analysis of these genes (Fig. 3b-c).

### Immune cells

**Fig. 4:**
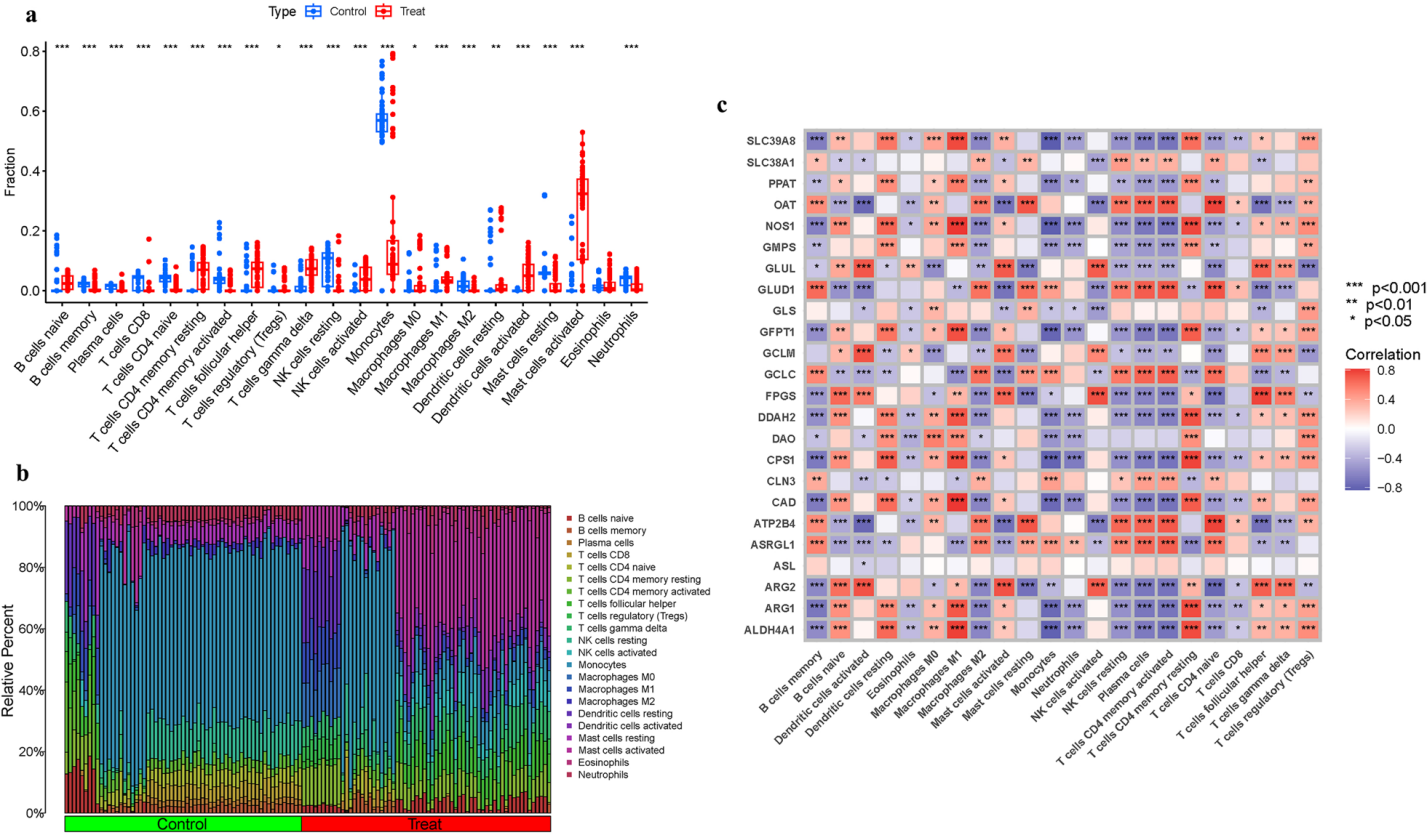
Expression of Immune cells. **a**–**b** Expression of immune cells in different clusters. **c** Correlation between GlnMgs and immune cells

The immunological environment has a critical role in the initiation and progression of OP. We created a barplot and a corplot to display the outcomes of immune cells (Fig. 4a, b).We also performed a correlation study of these genes and immune cells (Fig. 4c).

### Cluster analysis

**Fig. 5:**
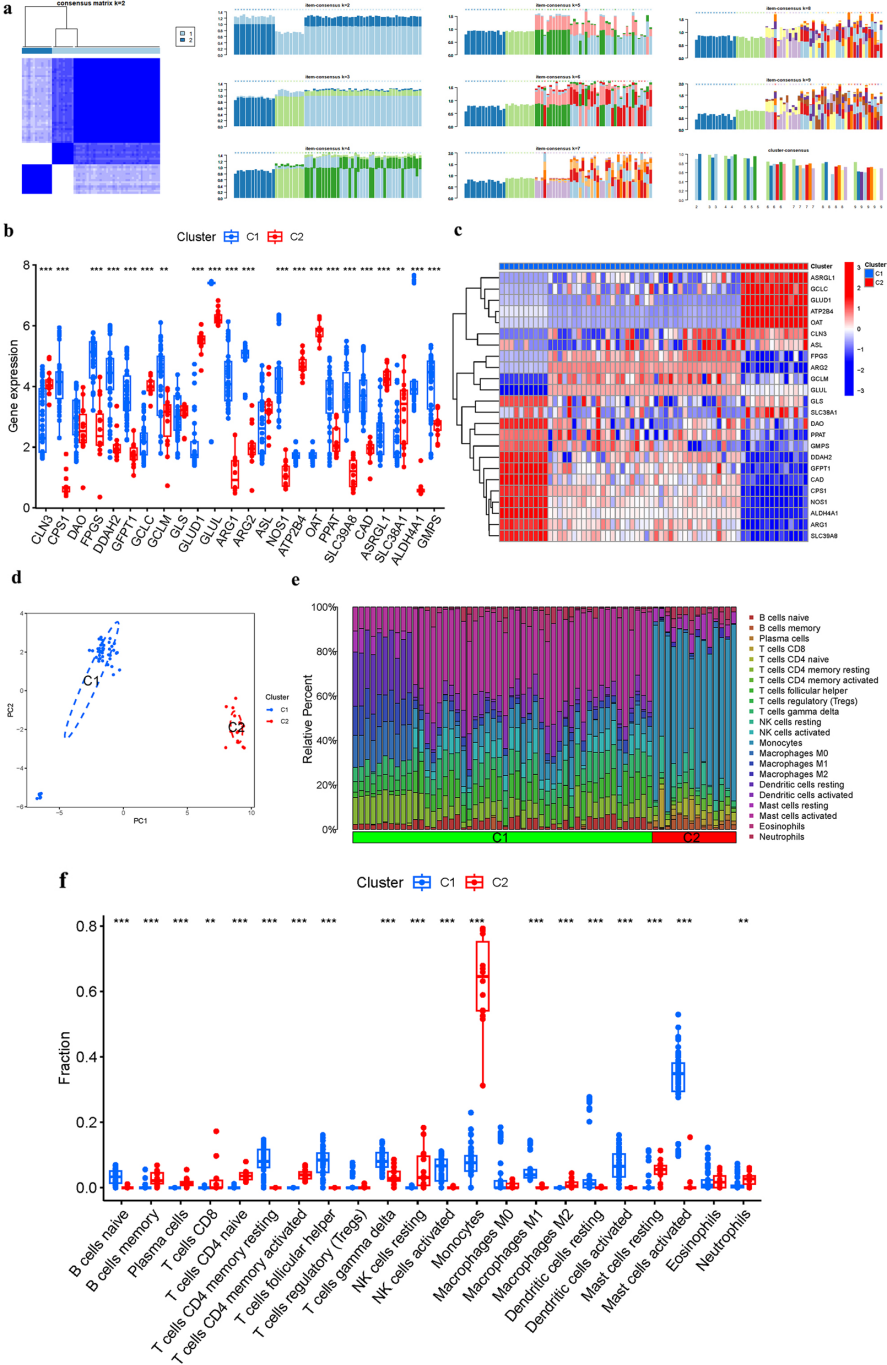
Cluster analysis. **a** Consensus. **b**–**c** GlnMgs in clusters. **d** PCA. **e**–**f** Immune cell infiltration

When *k* was 2, the intragroup correlations were the greatest, showing that GlnMgs may be used to separate OP patients into two groups (Fig. 5a). We also addressed the expression of the GlnMgs in distinct clusters based on this cluster. DAO, GLS, and ASL levels did not differ substantially in two groups (Fig. 5b, c). PCA shows that patients with varying risks were divided into two groups (Fig. 5d). We also examined the outcomes of immune cell infiltration by cluster (Fig. 5e, f).

### Analysis of functional enrichments

**Fig. 6:**
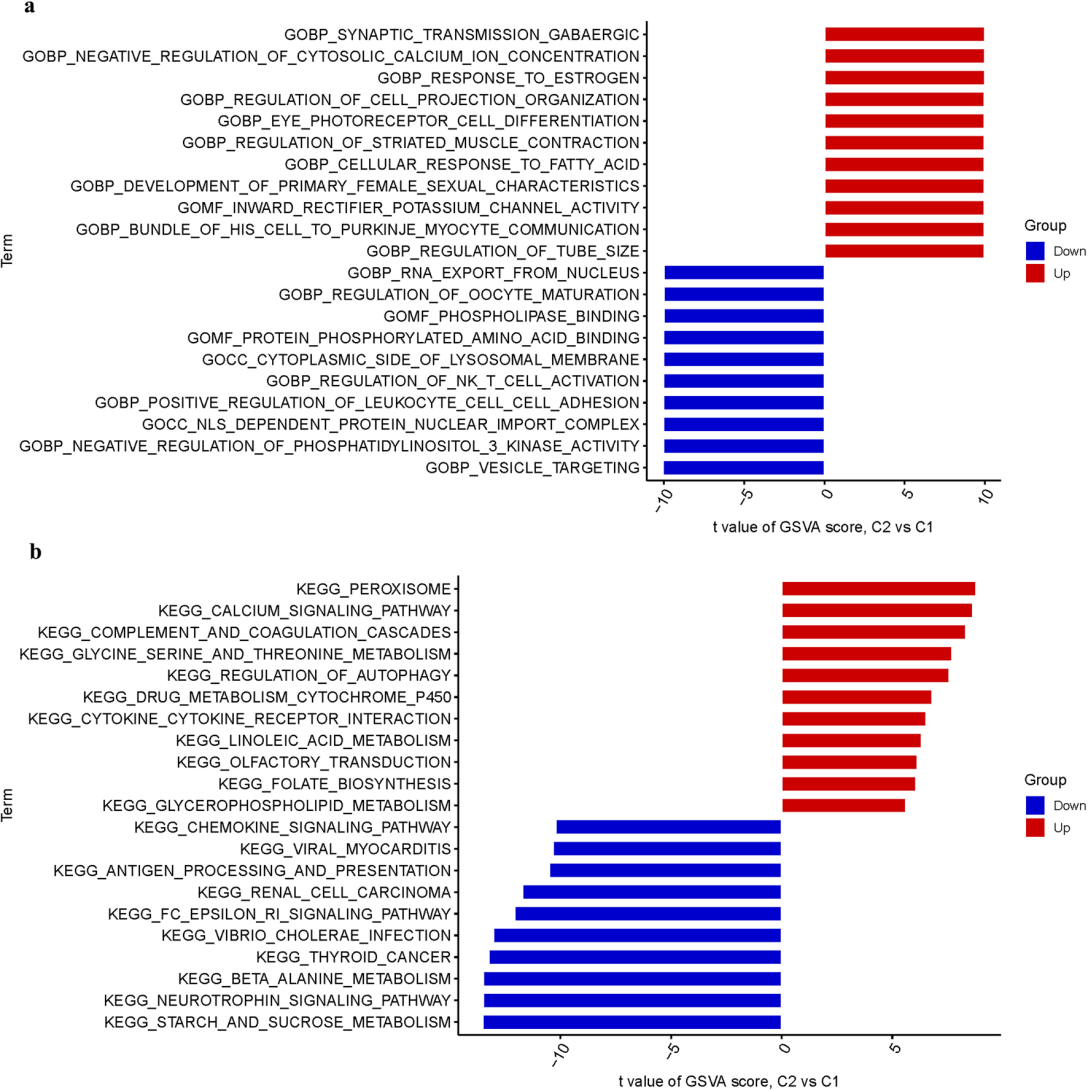
Enrichment analysis for DEGs. **a** GO. **b** KEGG

The enrichment analysis was carried out using the GlnMgs. The MF is primarily responsible for inward rectifier potassium channel activity, phospholipase binding; The BP primarily regulates synaptic transmission gabaergic, negative regulation of cytosolic calcium ion concentration; The CC primarily regulates cytoplasmic side of lysosomal membrane, nls dependent protein nuclear import complex (Fig. 6a). The examination of pathways revealed that the Peroxisome, calcium signaling pathway, and complement and coagulation cascades were enriched (Fig. 6b).

### Co-expression network

**Fig. 7:**
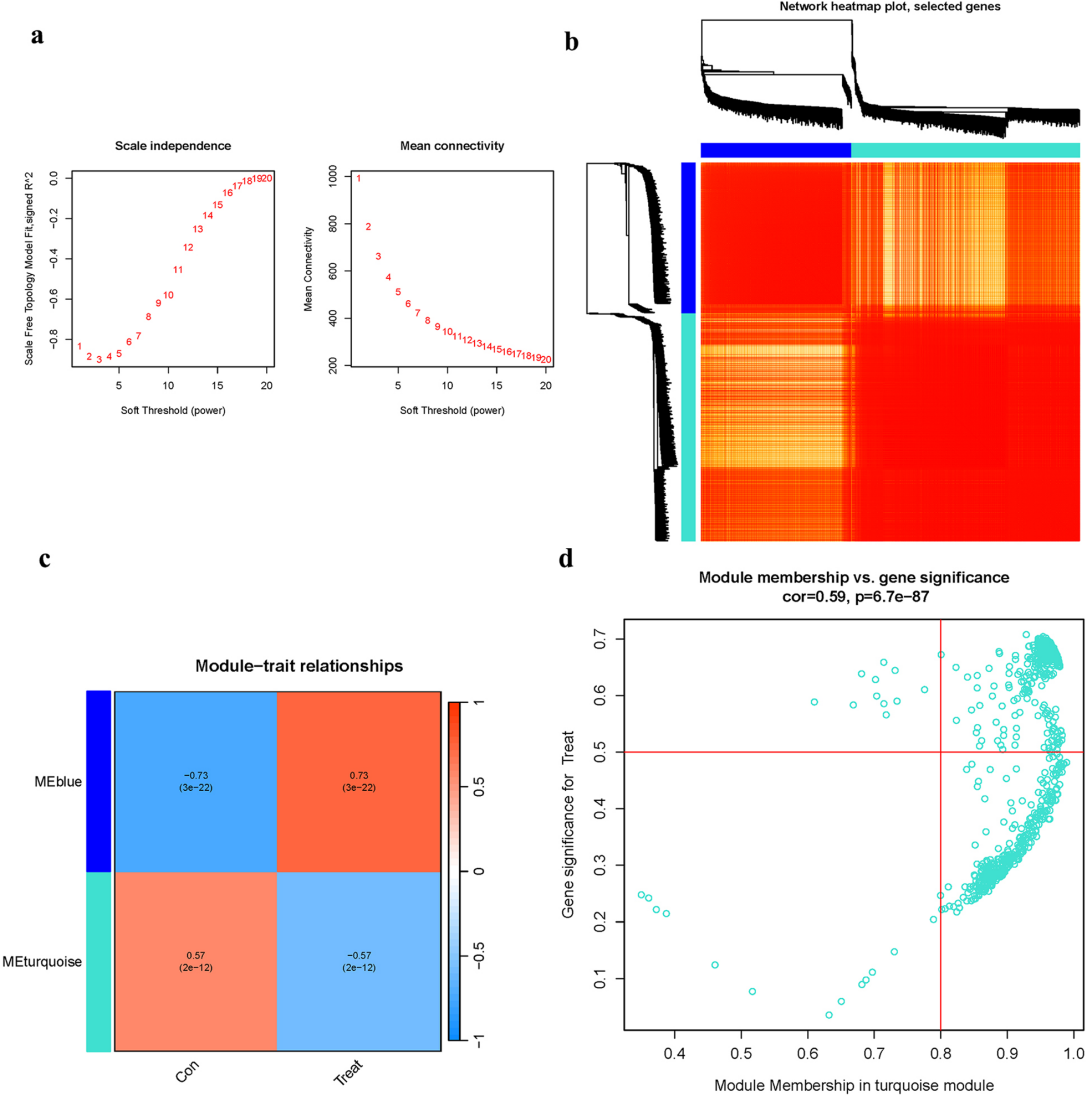
Co-expression module. **a** Index analysis. **b** Dendrogram clustering. **c** Heatmap. **d** Scatterplot

A soft-thresholding power was used to build an approximation scale-free topology for the network (Fig. 7a). The most variable genes were aggregated and integrated into two co-expression modules (Fig. 7b). Pearson's correlation analysis was investigated (Fig. 7c). The turquoise module was shown to be highly connected with the “Group” attribute (i.e., OP and Control) (Fig. 7d).

### Clustering co-expression network

**Fig. 8:**
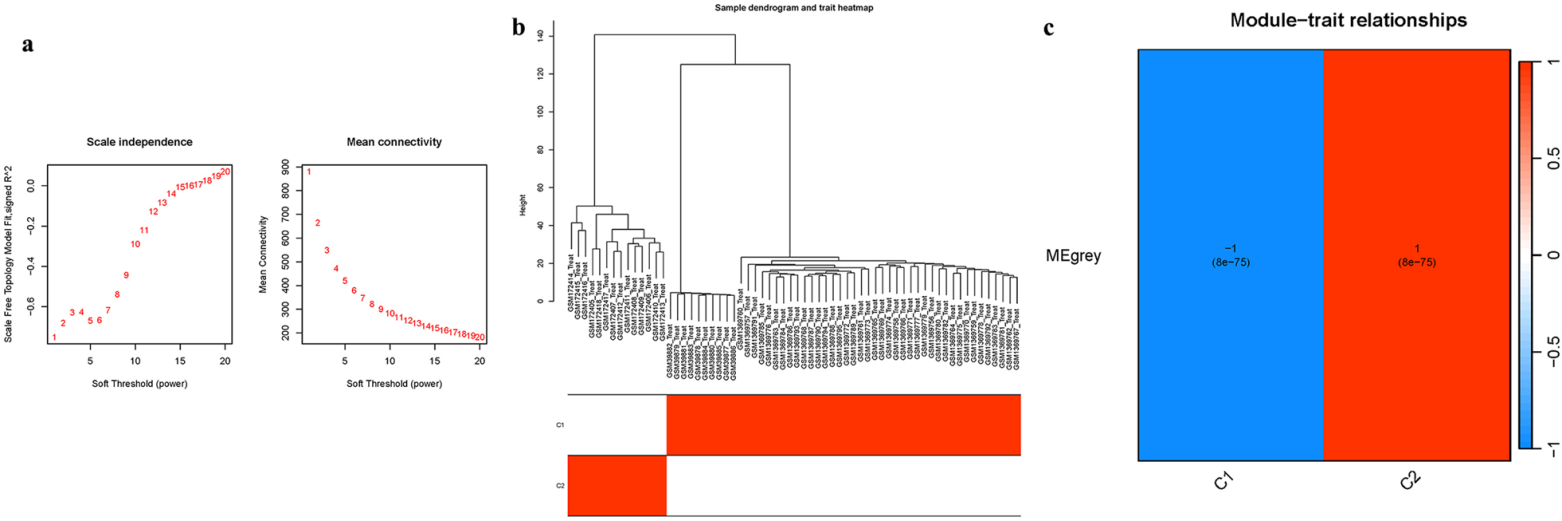
Cluster co-expression modules **a** Index analysis. **b** Dendrogram clustering **c** Heatmap

This research also builded an network (Fig. 8a). Clustering the variance genes resulted in the formation of co-expression modules (Fig. 8b). The link between module eigengene and clinical features was investigated. The module was shown to be strongly linked with the "Group" characteristic (i.e., OP and Control) (Fig. 8c).

### Construction of the model

**Fig. 9:**
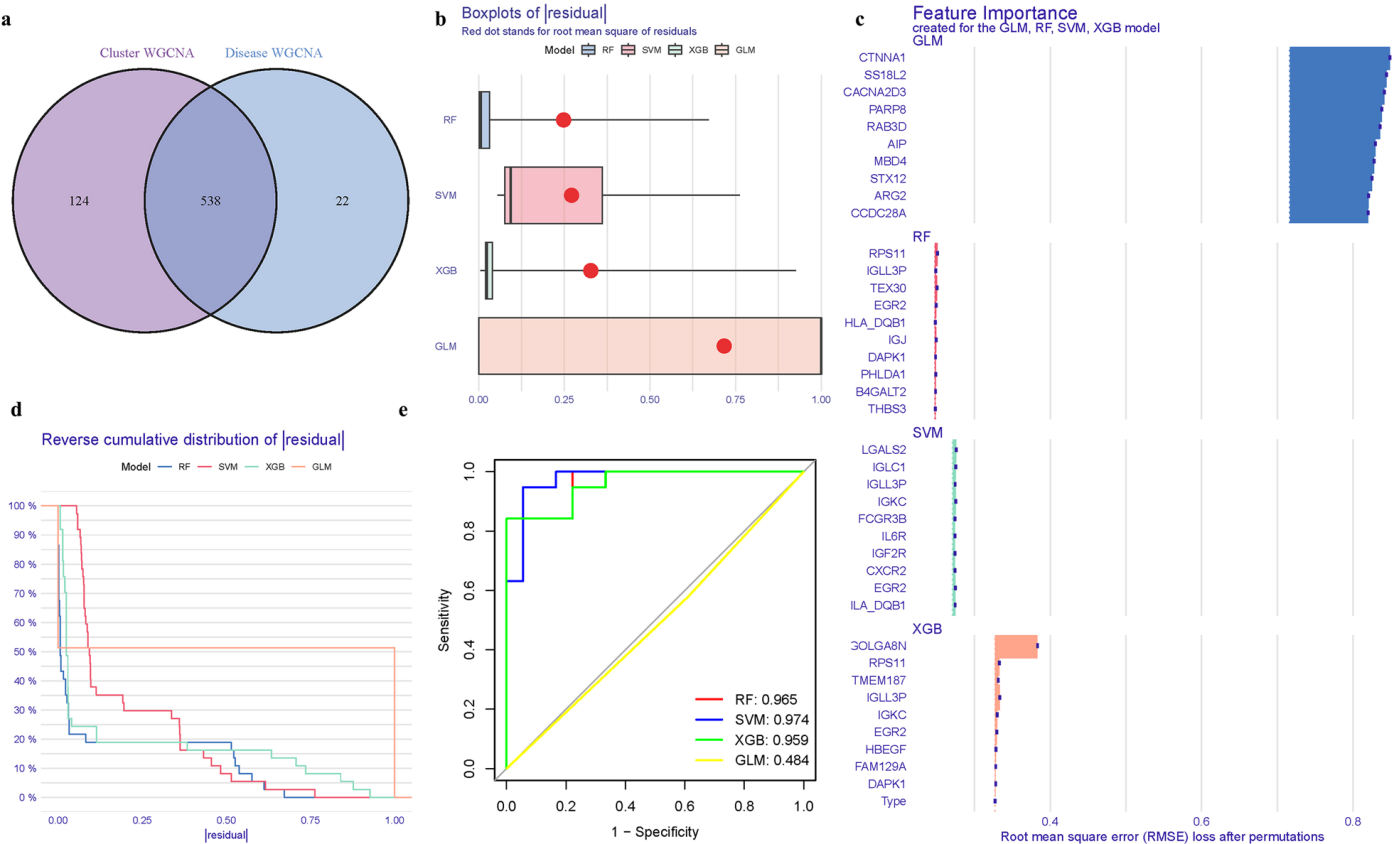
Model. **a** Venn. **b**–**c** Residual expression patterns. **d** Model trend chart. **e** AUC of train group

DEGs, turquoise module genes, and GlnMgs all overlap. Some genes were crossed (Fig. 9a) (Additional file 1: Table S5). The residual expression patterns of these genes in OP were represented using Boxplots (Fig. 9b).The proportions of the four distinct modes varied somewhat (Fig. 9c). Differences in the expression of the four models' predictive values at different phases (Fig. 9d). The diagnostic power of the GlnMgs in separating OP from control samples demonstrated an acceptable diagnostic value, with AUCs of RF: 0.965, SVM: 0.974, XGB: 0.959, GLM: 0.484 (Fig. 9e). The XGB model is clearly the most precise and steady.

### Model validation

**Fig. 10:**
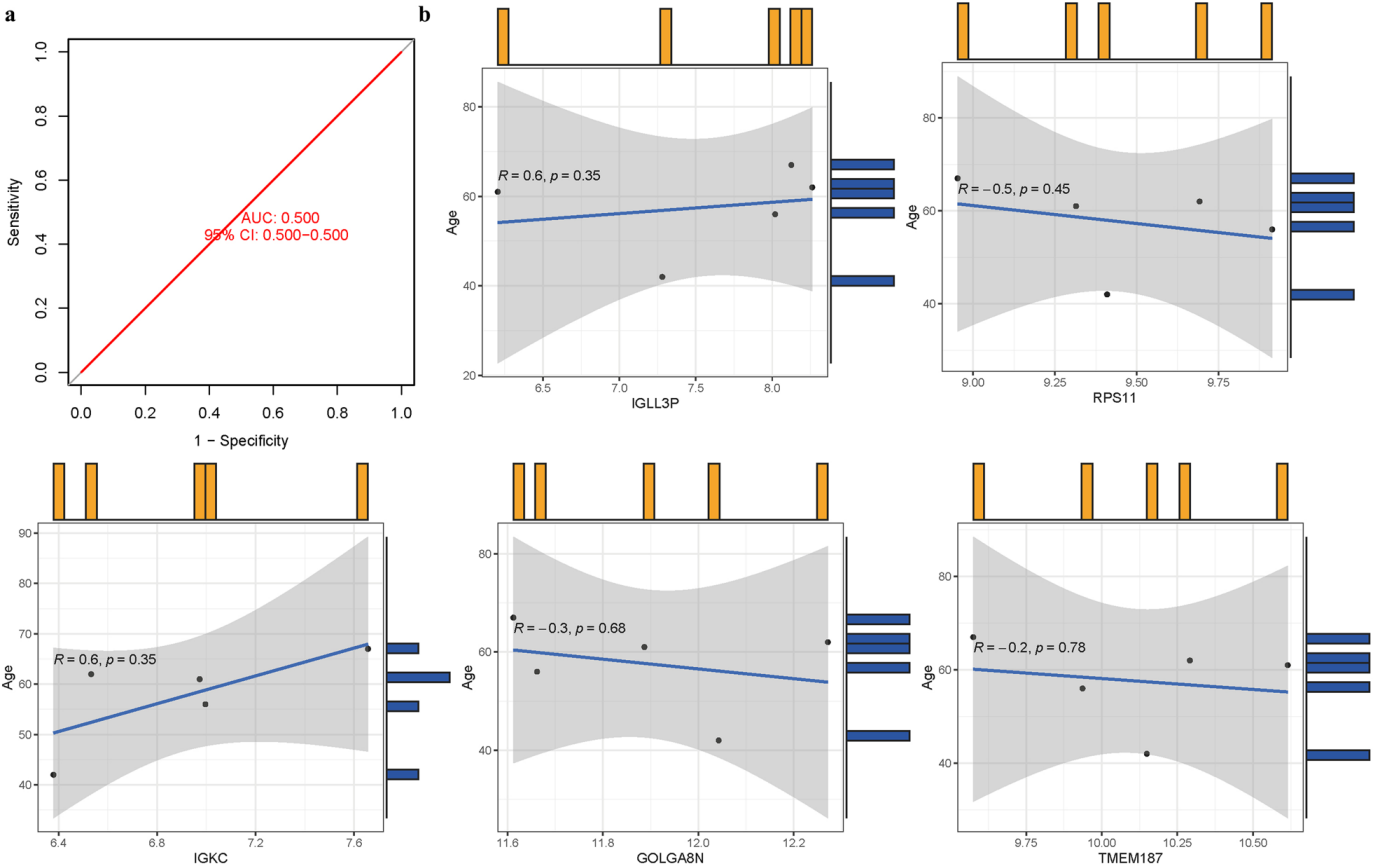
**a** AUC of test group. **b** Analysis of the relationship between hub genes and age

An AUC of 0.500 (95% CI 0.500–0.500) in GSE35956 (Fig. 10a). We also calculated the information of these five hub genes with age. GOLGA8N, RPS11, and TMEM187 were negatively correlated with age, while IGKC and IGLL3P were positively correlated with age. However, it should be noted that the P values of these genes were all greater than 0.05 (Fig. 10b) (Additional file 1: Table S6).

#### Drug-gene interactions

The all interacting genes were used for drug prediction (Additional file 1: Table S7).

## Discussions

OP is a prevalent systemic bone disease characterized by diminished bone density and mass, compromised bone microstructure, heightened bone fragility, and an elevated risk of fractures for diverse etiological factors [16]. Notably, one in every three women and one in every five men over the age of 50 face the susceptibility of osteoporotic fractures. Until the occurrence of a fracture or fractures, OP may remain subclinical. These fractures exert substantial physical and personal burdens on affected individuals, in addition to imposing a significant economic toll [17]. Apart from utilizing glucose, proliferating cancer cells also heavily rely on Gln as an indispensable energy and building block source. In fact, many tumor cells are so reliant on exogenous Gln that its absence results in their demise [18]. In light of the growing understanding and exploration of tumor biology, an increasing number of researchers have shifted their attention toward non-tumor aspects, recognizing the significance of investigating Gln metabolism and its potential implications beyond oncological contexts.

Gln is one of the most abundant nonessential amino acids (amino acids produced by the human body but not necessary in the diet) in circulation, contributing to nearly every biosynthetic pathway in proliferating cells [19]. It also acts as a nitrogen donor in the purine and pyrimidine synthesis, as well as a precursor in the formation of protein and glutathione. Because Gln-derived-KG feeds the TCA cycle, cancer cells can employ glutaminolysis to sustain the production of a variety of essential compounds [20]. Gln metabolism failure has been associated with cancer formation in several studies, and Gln metabolism-targeting drugs have been approved for a variety of malignancies. Metabolic needs and phenotypes may emerge when cancer progresses from premalignant lesions to clinically visible tumors to metastatic malignancies. Gln metabolism is gaining attention as an intriguing regulatory node that varies in a variety of clinical settings. Gln, the most common nonessential amino acid in circulation, is involved in a variety of cellular metabolic functions [21]. Glutaminase is an enzyme that deaminates gln to produce glutamate, a key intermediate metabolite with multiple metabolic uses in the cell [22]. A number of recent studies have emphasized the function of GlnMgs in a variety of aging-related diseases. Dai et al. [23], for example, looked into the possible functions of Gln-metabolism-related genes in hepatocellular carcinoma, while Liu et al. [24] established a Gln-metabolism signature for lung adenocarcinoma prognosis. The physiological importance of Gln metabolism in OP development is unknown.

In the context of OP, our study identified 24 DEGs associated with Gln. Leveraging a comprehensive approach, we utilized the intersection of DEGs, WGCNA, and GlnMgs to uncover these Gln DEGs, deepening our understanding of their involvement in OP. Furthermore, employing LASSO regression, we identified five hub GlnMgs (IGKC, TMEM187, RPS11, IGLL3P, GOLGA8N), and their diagnostic capacity was validated using external datasets, suggesting their potential implication in the pathogenesis of OP. Nevertheless, the mechanisms through which these genes may be associated with the regulation of specific transcription factors involved in Gln management remain to be established. Among the hub genes, IGKC represents an immunoglobulin, and extensive research has highlighted its impact on cancer development and immune-related factors when expressed at high levels [25, 26]. TMEM187 and SYTL4 genes have been found to interact directly with known autism spectrum disorder genes, with their mRNAs present in extracellular vesicles in the nervous system, facilitating the translation of active proteins in target cells [27]. This evidence suggests the potential candidacy of these genes for further exploration in the context of autism research, bolstering the validity and plausibility of our findings, as these Gln DEGs have shown links to malignancy processes in individuals with OP. Additionally, our study identified that a Gln-related trait, as observed in the GSE35956 research, may serve as an effective prognostic predictor. However, it is noteworthy that only a limited number of studies have investigated the gene alterations associated with Gln in the context of osteoporosis. Therefore, our research contributes valuable insights into this underexplored area, shedding light on potential avenues for further investigations and advancing our understanding of the role of Gln in osteoporosis pathogenesis.

Bone homeostasis relies on a dynamic equilibrium between bone production and resorption, intricately governed by a complex cytokine network [28]. The interplay between immune and bone cells at the immunoskeletal interface plays a pivotal role in regulating bone turnover, both in normal physiological conditions and pathological settings [29]. Notably, numerous diseases contributing to osteoporosis exhibit a chronic inflammatory background. Menopausal estrogen decline and the aging process foster osteoporosis by promoting the generation of osteoclastogenic inflammatory cytokines [30]. Inflammatory rheumatic disorders exemplify the tight interconnection between the immune system and bone, leading to local and systemic bone loss, driven by osteoclast hyperactivation and uncoupling of bone production and resorption [31, 32]. Building upon our previous investigations, this study also explores the expression patterns of GlnMgs in the immunological microenvironment. The examination of GlnMgs reveals substantial expression of B cells naive, T cells CD4 memory resting, and T cells follicular helper activated in cluster 1. Conversely, cluster 2 exhibits substantial expression of B cells memory, NK cells resting, and Monocytes. These findings underscore the intricate link between the pathophysiology of GlnMgs in osteoporosis and the underlying inflammatory and immunological responses. By unraveling the complex interactions between glutamine metabolism and the immune system, our research aims to provide valuable insights into the pathogenesis of osteoporosis, paving the way for potential therapeutic approaches targeting inflammation and immunomodulation to address this prevalent bone disorder.

The investigation of biomarkers in the context of OP has remained relatively understudied. Recently, bioinformatics analyses have emerged as a valuable tool to explore the intricate link between metabolism and OP [33–35]. Mo et al. conducted a comprehensive study and identified six potential biomarkers (COL1A1, IBSP, CTSP, CTSD, RAC2, MAF, and THBS1) associated with OP through the construction of a robust model. Similarly, Liu et al. discovered that PRKCB, GSDMD, ARMCX3, and CASP3 are hub genes with potential as molecular targets for OP prognosis and therapy. Notably, investigations pertaining to Gln and its relevance to OP are lacking in the current literature. Our novel research approach, based on cell metabolism, seeks to uncover effective therapeutic methods for OP. Distinguished from prior studies, our research incorporates a unique and innovative technique, leveraging an extensive dataset of GlnMgs from GEO. Although providing a solid theoretical foundation and research framework, this study also faces several limitations. One key challenge lies in comprehending the intricate underlying systems that govern Gln's influence on OP. Both in vivo and in vitro experiments hold promise in this context; however, their outcomes have generated new areas of investigation, warranting further exploration. Furthermore, the relationship between prognostic genes and Gln remains elusive, holding the potential to shed light on the involvement of GlnMgs in OP pathogenesis. By addressing this knowledge gap, our research endeavors to contribute valuable insights into the molecular mechanisms underlying OP and its potential therapeutic avenues.

## Conclusions

OP develops and progresses due to interactions between various targets, routes, signaling pathways, and mechanisms, and the regulation process is synergistic and bi-directional. GlnMgs influence IGKC, TMEM187, RPS11, IGLL3P, GOLGA8N synthesis, which can activate or inhibit the calcium, Chemokine, and Epsilon ri signaling pathway. The following suggestions for future improvements are made: (1) The number of data sources will be increased in the future. (2) Additional researches will be carried out to establish whether effective medicines might improve the Bone, bone resorption and bone destruction balance of OP by modulating these GlnMgs.

### Supplementary Information

**Additional file 1**. Supplementary Tables.

## Data Availability

The datasets generated during and/or analyzed during the current study are available in the appendix.

## Abbreviations

OP: Osteoporosis
GO: Gene Ontology
TCM: Traditional Chinese medicine
MF: Molecular functions
KEGG: Kyoto encyclopedia of genes and genomes
GEO: Gene expression omnibus
GlnMgs: Gln-metabolism genes
BP: Biological processes
CC: Cellular components
DEGs: Differentially expressed genes
