# Supplementary material for: Bioinformatics and machine learning were used to validate glutamine metabolism-related genes and immunotherapy in osteoporosis patients
Source: J Orthop Surg Res. 2023 Sep 14;18:685. doi: 10.1186/s13018-023-04152-2 (PMC10503203; doi:10.1186/s13018-023-04152-2)
Supplement: Supplementary file 1 — Additional file 1. Supplementary Tables. [file 13018_2023_4152_MOESM1_ESM.doc]

Bioinformatics and Machine Learning were used to Validate Glutamine Metabolism-Related genes and Immunotherapy in Osteoporosis Patients

**Supplementary appendix to the manuscript**

Contents of supplementary appendix

[Appendix 1 3](#__RefHeading___Toc31153)

[Datasets and Glutamine Metabolism 3](#__RefHeading___Toc1396)

[Table S1. Glutamine Metabolism genes 3](#__RefHeading___Toc5462)

[Appendix 2 4](#__RefHeading___Toc15712)

[DEGs linked to GlnMgs 4](#__RefHeading___Toc10913)

[Table S2. 24 DEGs linked to GlnMgs. 4](#__RefHeading___Toc11634)

[Appendix 3 6](#__RefHeading___Toc5429)

[Table S3. chromosomal positions of GlnMgs. 6](#__RefHeading___Toc23929)

[Appendix 4 9](#__RefHeading___Toc21877)

[InterGenes 9](#__RefHeading___Toc19672)

[Table S4. InterGenes. 9](#__RefHeading___Toc5020)

[Appendix 5 13](#__RefHeading___Toc6070)

[ImportanceGene.XGB 13](#__RefHeading___Toc9381)

[Table S5. ImportanceGene.XGB. 13](#__RefHeading___Toc2765)

[Appendix 6 14](#__RefHeading___Toc26471)

[corResult 14](#__RefHeading___Toc17045)

[Table S6. corResult. 14](#__RefHeading___Toc17290)

[Appendix 7 15](#__RefHeading___Toc6363)

[Table S7. Drug prediction. 15](#__RefHeading___Toc8522)

# Appendix 1

**Datasets and Glutamine Metabolism**

**Table S1. Glutamine Metabolism genes**

| GLYATL1B | PHGDH | ASL | PRODH2 | GAD2 |
| --- | --- | --- | --- | --- |
| MTHFS | GFPT1 | ASNS | SLC39A8 | MECP2 |
| FTCD | GGT1 | ASS1 | PYCR3 | ALDH18A1 |
| CLN3 | GLS2 | NOS1 | TAT | GAD1 |
| NOXRED1 | GCLC | NOS2 | CAD | ART4 |
| UROC1 | GCLM | NOS3 | ALDH5A1 | PYCR1 |
| CPS1 | GLS | ATP2B4 | AGMAT | NR1H4 |
| ADHFE1 | GLUD1 | OAT | DGLUCY | SLC7A11 |
| AMDHD1 | GLUD2 | OTC | ASRGL1 | MIR21 |
| CTPS1 | GLUL | AADAT | SLC38A1 | RIMKLB |
| DAO | GOT1 | LGSN | ATCAY | GFPT2 |
| NAGS | GOT2 | PFAS | SLC25A12 | DDAH1 |
| FAH | RIMKLA | ASNSD1 | ALDH4A1 | ARG2 |
| SIRT4 | PYCR2 | PPAT | GMPS | NIT2 |
| FPGS | HAL | PRODH | ARHGAP11B | GLYATL1 |
| DDAH2 | ARG1 | CTPS2 | SLC7A7 |  |

# Appendix 2

## **DEGs linked to GlnMgs**

**Table S2. 24 DEGs linked to** **GlnMgs.**

| ID | GSM40049 | GSM40052 | GSM40202 | GSM40204 | GSM40208 | GSM40223 |
| --- | --- | --- | --- | --- | --- | --- |
| CLN3 | 2.268645 | 2.27589 | 2.271669 | 2.272345 | 2.27302 | 2.272345 |
| CPS1 | 5.987745 | 5.924696 | 5.91186 | 6.04484 | 6.099229 | 5.864309 |
| DAO | 3.908285 | 4.029361 | 3.977261 | 3.956101 | 3.922265 | 3.999384 |
| FPGS | 3.931961 | 3.91422 | 3.896962 | 3.837649 | 4.000139 | 3.954792 |
| DDAH2 | 6.004758 | 5.857145 | 5.937144 | 5.951215 | 6.071571 | 5.976128 |
| GFPT1 | 5.164564 | 5.083215 | 5.143215 | 5.119983 | 5.049261 | 5.148652 |
| GCLC | 2.268645 | 2.27589 | 2.271669 | 2.272345 | 2.27302 | 2.272345 |
| GCLM | 2.268645 | 2.27589 | 2.271669 | 2.272345 | 2.27302 | 2.272345 |
| GLS | 3.643662 | 3.675385 | 3.706729 | 3.664418 | 3.681936 | 3.675385 |
| GLUD1 | 2.268645 | 2.27589 | 2.271669 | 2.272345 | 2.27302 | 2.272345 |
| GLUL | 2.268645 | 2.27589 | 2.271669 | 2.272345 | 2.27302 | 2.272345 |
| ARG1 | 5.369877 | 5.663851 | 5.797566 | 6.990518 | 5.493312 | 5.323677 |
| ARG2 | 3.885514 | 3.942652 | 3.801736 | 3.874907 | 3.85211 | 3.903203 |
| ASL | 2.268645 | 2.27589 | 2.271669 | 2.272345 | 2.27302 | 2.272345 |
| NOS1 | 6.360464 | 6.438453 | 6.241643 | 6.355209 | 6.502188 | 6.408545 |
| ATP2B4 | 2.268645 | 2.27589 | 2.271669 | 2.272345 | 2.27302 | 2.272345 |
| OAT | 2.268645 | 2.27589 | 2.271669 | 2.272345 | 2.27302 | 2.272345 |
| PPAT | 4.352376 | 4.282826 | 4.375564 | 4.278802 | 4.255238 | 4.274033 |
| SLC39A8 | 5.276964 | 5.199002 | 5.73961 | 5.688804 | 5.177887 | 5.201673 |
| CAD | 5.080698 | 5.163256 | 5.121294 | 5.167224 | 5.248144 | 5.168434 |
| ASRGL1 | 2.268645 | 2.27589 | 2.271669 | 2.272345 | 2.27302 | 2.272345 |
| SLC38A1 | 2.268645 | 2.27589 | 2.271669 | 2.272345 | 2.27302 | 2.272345 |
| ALDH4A1 | 7.79581 | 7.633483 | 7.633483 | 7.601011 | 7.727708 | 7.79581 |
| GMPS | 5.049261 | 4.986843 | 5.091087 | 5.157765 | 4.897895 | 4.861031 |

# Appendix 3

**Chromosomal positions of GlnMgs**

**Table S3. chromosomal positions of GlnMgs.**

| Chromosome | chromStart | chromEnd | Gene |
| --- | --- | --- | --- |
| chr1 | 15572353 | 15585110 | AGMAT |
| chr1 | 18871430 | 18902781 | ALDH4A1 |
| chr1 | 40979335 | 41012565 | CTPS1 |
| chr1 | 42380795 | 42422578 | RIMKLA |
| chr1 | 85318481 | 85578363 | DDAH1 |
| chr1 | 93885205 | 93909456 | GCLM |
| chr1 | 119659798 | 119744215 | PHGDH |
| chr1 | 182381704 | 182392206 | GLUL |
| chr1 | 203626561 | 203744081 | ATP2B4 |
| chr1 | 225919877 | 225924340 | PYCR2 |
| chr2 | 27217390 | 27243943 | CAD |
| chr2 | 69319769 | 69387254 | GFPT1 |
| chr2 | 170813213 | 170861151 | GAD1 |
| chr2 | 171784370 | 171999859 | SLC25A12 |
| chr2 | 189661385 | 189670831 | ASNSD1 |
| chr2 | 190880827 | 190965552 | GLS |
| chr2 | 210477682 | 210679107 | CPS1 |
| chr3 | 100334701 | 100361635 | NIT2 |
| chr3 | 126481281 | 126517773 | UROC1 |
| chr3 | 155870536 | 155944026 | GMPS |
| chr4 | 56393362 | 56435615 | PPAT |
| chr4 | 102251041 | 102431258 | SLC39A8 |
| chr4 | 138164097 | 138242349 | SLC7A11 |
| chr4 | 170060222 | 170091699 | AADAT |
| chr5 | 180300690 | 180353387 | GFPT2 |
| chr6 | 24494852 | 24537207 | ALDH5A1 |
| chr6 | 31727038 | 31730617 | DDAH2 |
| chr6 | 53497341 | 53616970 | GCLC |
| chr6 | 63275951 | 63319977 | LGSN |
| chr6 | 131573144 | 131584332 | ARG1 |
| chr7 | 66075798 | 66093558 | ASL |
| chr7 | 97852118 | 97872542 | ASNS |
| chr7 | 150990995 | 151014588 | NOS3 |
| chr8 | 66430185 | 66471601 | ADHFE1 |
| chr9 | 127794597 | 127814327 | FPGS |
| chr9 | 130444929 | 130501274 | ASS1 |
| chr10 | 26216307 | 26304558 | GAD2 |
| chr10 | 87050486 | 87094866 | GLUD1 |
| chr10 | 95605929 | 95656706 | ALDH18A1 |
| chr10 | 99396870 | 99430624 | GOT1 |
| chr10 | 124397303 | 124418976 | OAT |
| chr11 | 58905398 | 59043527 | GLYATL1 |
| chr11 | 62337448 | 62393412 | ASRGL1 |
| chr12 | 8681600 | 8783095 | RIMKLB |
| chr12 | 14825569 | 14843495 | ART4 |
| chr12 | 46183063 | 46270017 | SLC38A1 |
| chr12 | 56470944 | 56488414 | GLS2 |
| chr12 | 95943293 | 95968716 | AMDHD1 |
| chr12 | 95972662 | 95996365 | HAL |
| chr12 | 100473708 | 100564413 | NR1H4 |
| chr12 | 108858932 | 108901043 | DAO |
| chr12 | 117208142 | 117452170 | NOS1 |
| chr12 | 120302316 | 120313249 | SIRT4 |
| chr14 | 22773222 | 22829820 | SLC7A7 |
| chr14 | 67619798 | 67651720 | ARG2 |
| chr14 | 77394021 | 77423517 | NOXRED1 |
| chr15 | 30624494 | 30772993 | ARHGAP11B |
| chr15 | 79833585 | 79897379 | MTHFS |
| chr15 | 80152490 | 80186946 | FAH |
| chr16 | 28477279 | 28495575 | CLN3 |
| chr16 | 58707131 | 58734357 | GOT2 |
| chr16 | 71565660 | 71577130 | TAT |
| chr17 | 8247618 | 8270491 | PFAS |
| chr17 | 27756766 | 27800499 | NOS2 |
| chr17 | 44004546 | 44009063 | NAGS |
| chr17 | 81932384 | 81942412 | PYCR1 |
| chr19 | 3879864 | 3928079 | ATCAY |
| chr19 | 35799988 | 35813299 | PRODH2 |
| chr21 | 46136262 | 46155567 | FTCD |
| chr22 | 18912777 | 18936553 | PRODH |
| chr22 | 24583750 | 24629005 | GGT1 |
| chrX | 16588003 | 16712936 | CTPS2 |
| chrX | 38352545 | 38421450 | OTC |
| chrX | 121047588 | 121050080 | GLUD2 |
| chrX | 154021573 | 154137103 | MECP2 |

# Appendix 4

**InterGenes**

**Table S4. InterGenes.**

| DAPK1 | FHL1 | DUT | CUTA | SUB1 | MYL6 |
| --- | --- | --- | --- | --- | --- |
| UNC50 | ABCF3 | IGLL3P | RNF44 | RAC1 | Y16709 |
| WASF2 | STAU1 | TINF2 | TNFAIP8 | CCR2 | HLA-DPB1 |
| MLF2 | RASSF4 | DDX39A | PUM1 | FYN | HUWE1 |
| TMEM187 | LSM7 | ATOX1 | LILRA2 | MRPL33 | RPL18 |
| IQGAP2 | CLINT1 | GLTP | RBL2 | SLC38A2 | TUBA1B |
| FAM134B | CITED2 | XBP1 | GNB2 | LBR | PLBD1 |
| TSPYL2 | YY1AP1 | HADHA | UBE4A | STARD7 | ANXA2 |
| BTG3 | FDFT1 | BASP1 | CCT8 | TPI1 | IFITM2 |
| PUF60 | PAQR6 | YY1 | PSMB4 | TPST2 | TUBA1C |
| MBD4 | OGT | IL6R | TMED2 | NONO | HLA-DPA1 |
| ABCG4 | KLK1 | CRIP1 | WRB | NDUFA1 | S100A11 |
| SMS | THBS3 | NCKAP1L | RBM3 | EEF1D | RPS27A |
| OSTF1 | CETN1 | RPN1 | STAT6 | HMGB2 | KCTD12 |
| EPHA2 | RNF4 | ESD | TRAPPC8 | ARRB2 | RPS21 |
| TMPRSS11D | TLR1 | FAM129A | PNN | ICAM3 | RPL31 |
| NPY | CTRC | NDUFA13 | EIF4B | SF3B1 | SH3BGRL3 |
| SPHK1 | INTS8 | TES | TIMP2 | LGALS2 | MYL12B |
| CCDC28A | VWF | SNRPB | ANP32A | MYH9 | LGALS1 |
| TEX30 | PSMC5 | MDH2 | PSMA2 | CD93 | PFN1 |
| SLC6A4 | BTBD1 | SRSF3 | HNRNPC | GSTP1 | RPL37A |
| RP11-10N23.4 | EIF2B1 | SRPR | CAST | PGK1 | RPL12 |
| HTR6 | IL1RAPL2 | CHP1 | RALB | RTN3 | HLA-G |
| S100A2 | GANAB | SH3BP5 | ATP5G3 | CFP | ARPC2 |
| CACNA2D3 | C1QBP | TAF7 | ATP5G2 | PPA1 | NACA |
| IGKC | MBD2 | SERINC5 | CAPG | EIF4H | S100A12 |
| CDC37 | ACAA1 | LAT2 | PCBP2 | ATP5J | EEF2 |
| TCF25 | CNOT8 | MGAT1 | EFHD2 | CSF3R | RPLP0 |
| SLC9A3R1 | ENOPH1 | FCGR3B | CANX | H2AFY | RPL24 |
| GCHFR | RNF146 | RAB1B | CDIPT | SORL1 | TXNIP |
| SPSB3 | PPM1B | NCL | HK3 | HLA-DMA | RPS25 |
| CNPY3 | RARRES3 | LMO2 | RXRA | PPIB | CTSS |
| TBCB | NDUFV2 | GRB2 | GNAI2 | CD1D | RPL6 |
| STX12 | MICB | LILRA6 | CERS2 | CAPN2 | RPS10 |
| IST1 | RNPS1 | CDV3 | RAP1A | CYBA | S100A8 |
| LRMP | SEPW1 | MAN2B1 | SRSF7 | KARS | HLA-C |
| FAM134C | RPS6KA3 | SYK | SELPLG | IFNGR1 | LYZ |
| PIN1 | CCDC71 | UBE2NL | GYG1 | CAPZB | PPBP |
| ZNF106 | MEA1 | FRAT2 | PCMT1 | APLP2 | SIRT1 |
| KRT10 | IGF2R | CBX4 | BST1 | TLR8 | TMEM9B |
| CASP8 | SNRPD3 | SF3B4 | HNRNPR | ANXA2P2 | LUC7L3 |
| TULP2 | PRPF4B | EGR2 | HDAC1 | SSR4 | EIF6 |
| EXTL3 | SDF4 | DUSP22 | GIMAP6 | SEC22B | POLR1D |
| IL3RA | MARCO | QPCT | SUMO4 | H2AFZ | GNB5 |
| PLK3 | RTN1 | ARL6IP1 | HSP90AB1 | ALOX5AP | CDK5 |
| NR1H3 | GLUD1 | HNRNPD | CXCR4 | SNRPD2 | LITAF |
| RAB6B | VPS51 | BANF1 | ATP5I | RPL36AL | SCAF8 |
| GS1-111G14.1 | POLR2L | UBE2N | PKM | IFITM1 | RPS6KA1 |
| CLUH | CD4 | NOTCH2 | CDK5RAP3 | RAB8A | KPNB1 |
| RAB3D | M6PR | FLI1 | SON | RPL35A | PRPF8 |
| SEC23B | CXCR2 | MYCBP2 | ABHD17A | IQGAP1 | LILRA1 |
| CTNNA1 | SNRNP27 | DEGS1 | CDC42 | CST3 | NDUFB8 |
| CIDEB | RPA2 | LSM1 | COX5B | RNASE2 | HPCAL1 |
| IRF2BP1 | MAP3K5 | CTDSP2 | FBL | PTPN6 | IL2RG |
| AIP | RPS11 | HP1BP3 | DPM1 | GIMAP4 | CRBN |
| ARG2 | PRPF40A | ATP6V1E1 | RAB27A | TPP1 | TBC1D9 |
| KDELR2 | BAG6 | SEC61G | LSP1 | XIST | CLTA |
| LYZL6 | HBEGF | CNN2 | HMGN1 | C14orf2 | ARCN1 |
| PES1 | HSD17B10 | ARF6 | HMGN4 | COX4I1 | UQCRQ |
| SERPINA4 | HBD | NADK | FCER1A | PTPRO | CALHM2 |
| STRA6 | UBA1 | PDIA6 | GOLGA8N | CSF1R | HHEX |
| S100A1 | CSRP1 | DOK3 | DHX15 | NPM1 | CD33 |
| HNRNPA3 | RNF114 | RAN | DAD1 | RNASE6 | IRF9 |
| ETHE1 | KDELR1 | PRKCSH | SEPT9 | ADD3 | EDF1 |
| IRAK1 | PHLDA1 | MAPKAPK3 | ARHGEF6 | RHOG | PRKCD |
| CARTPT | LSM14A | NAGA | TOP2B | COX7C | KHDRBS1 |
| FAM189B | SRRM1 | EIF4A3 | DDX17 | FLNA | CTDSP1 |
| PARP8 | PSMA7 | TGOLN2 | PRR11 | CLC | ARF3 |
| IL1A | SAP18 | ARID1A | P4HB | CD164 | ITGAL |
| CD47 | KDM3B | HNRNPDL | GZMB | 15-Sep | SASH3 |
| LOC102723620 | FLII | ACADVL | EID1 | CX3CR1 | VASP |
| PARD3 | SFT2D2 | CTR9 | CD163 | CCNI | CD37 |
| NOD2 | PDHB | TMED10 | WIPF1 | MORF4L1 | GOLPH3 |
| GPR21 | UBE2L3 | CNIH1 | RNASET2 | IGLC1 | P2RY13 |
| PSMC6 | EPS15 | CD55 | FKBP5 | DPYSL2 | TMEM230 |
| UBE2G1 | SNW1 | CRTAP | ATP6V0E1 | FXYD5 | IL17RA |
| WDR11 | PSMD4 | OAT | HINT1 | PLP2 | VAMP3 |
| DNAJC1 | NDUFA3 | GPR65 | SLA | IGJ | PECAM1 |
| SLC15A3 | PTAFR | DYSF | GLIPR1 | PPP1CC | UCP2 |
| MPP2 | MYL12A | SIDT2 | ZNF217 | LST1 | RPL36 |
| ZBTB18 | METTL7A | DENND4B | RAD21 | ATP5L | CSF2RB |
| LEFTY1 | RAB8B | METTL9 | CYTIP | YWHAB | MYO1F |
| AIDA | PIK3CD | TMED9 | LAMP1 | RPS28 | RAC2 |
| CD81 | HLA-DQB1 | YWHAE | YWHAQ | ITGAM | DUSP6 |
| CECR5 | FNTA | SRSF11 | CSK | GDI2 | RPS14 |
| SS18L2 | CAB39 | AHNAK | EIF2S3 | JTB | RPS9 |
| IK | UBL5 | DCTN3 | ENO1 | RPL14 | CD52 |
| B4GALT2 | HMHA1 | MAPRE1 | PRNP | RPL15 | RPL38 |
| BRD2 | NPIPA1 | SERBP1 | GNS | HLA-F | HLA-J |
| PLXNC1 | CEBPA | TOB1 | DBI |  |  |

# Appendix 5

**ImportanceGene.XGB**

**Table S5. ImportanceGene.XGB.**

| variable | permutation | dropout_loss | label |
| --- | --- | --- | --- |
| IGKC | 0 | 0.329836334 | XGB |
| TMEM187 | 0 | 0.33142518 | XGB |
| RPS11 | 0 | 0.332909296 | XGB |
| IGLL3P | 0 | 0.333258659 | XGB |
| GOLGA8N | 0 | 0.383312463 | XGB |

# Appendix 6

**corResult**

**Table S6. corResult.**

| Gene | Clinical | cor | pvalue |
| --- | --- | --- | --- |
| IGKC | Age | 0.6 | 0.35 |
| TMEM187 | Age | -0.2 | 0.783333333 |
| RPS11 | Age | -0.5 | 0.45 |
| IGLL3P | Age | 0.6 | 0.35 |
| GOLGA8N | Age | -0.3 | 0.683333333 |

# Appendix 7

**Drug prediction**

**Table S7. Drug prediction.**

| search_term | match_term | gene | drug | interaction_types |
| --- | --- | --- | --- | --- |
| DAPK1 | DAPK1 | DAPK1 | GEMCITABINE |  |
| EPHA2 | EPHA2 | EPHA2 | SORAFENIB |  |
| EPHA2 | EPHA2 | EPHA2 | DASATINIB | inhibitor|antagonist |
| EPHA2 | EPHA2 | EPHA2 | VANDETANIB | inhibitor |
| EPHA2 | EPHA2 | EPHA2 | REGORAFENIB | inhibitor |
| NPY | NPY | NPY | BROMOCRIPTINE |  |
| NPY | NPY | NPY | HALOPERIDOL |  |
| SLC6A4 | SLC6A4 | SLC6A4 | LEVOMILNACIPRAN | inhibitor |
| SLC6A4 | SLC6A4 | SLC6A4 | AMOXAPINE | inhibitor |
| SLC6A4 | SLC6A4 | SLC6A4 | DAPOXETINE | inhibitor |
| SLC6A4 | SLC6A4 | SLC6A4 | SERTRALINE | negative modulator|binder|inhibitor |
| SLC6A4 | SLC6A4 | SLC6A4 | RISPERIDONE |  |
| SLC6A4 | SLC6A4 | SLC6A4 | QUETIAPINE |  |
| SLC6A4 | SLC6A4 | SLC6A4 | CLOMIPRAMINE | inhibitor |
| SLC6A4 | SLC6A4 | SLC6A4 | DESVENLAFAXINE | inhibitor |
| SLC6A4 | SLC6A4 | SLC6A4 | NEFAZODONE | inhibitor |
| SLC6A4 | SLC6A4 | SLC6A4 | AMITRIPTYLINE | inhibitor |
| SLC6A4 | SLC6A4 | SLC6A4 | FLUOXETINE | inhibitor |
| SLC6A4 | SLC6A4 | SLC6A4 | DULOXETINE | inhibitor |
| SLC6A4 | SLC6A4 | SLC6A4 | MORPHINE |  |
| SLC6A4 | SLC6A4 | SLC6A4 | DOXEPIN | inhibitor |
| SLC6A4 | SLC6A4 | SLC6A4 | TRAZODONE | inhibitor |
| SLC6A4 | SLC6A4 | SLC6A4 | CITALOPRAM | inhibitor |
| SLC6A4 | SLC6A4 | SLC6A4 | METHYLPHENIDATE | inhibitor |
| SLC6A4 | SLC6A4 | SLC6A4 | MILNACIPRAN | inhibitor |
| SLC6A4 | SLC6A4 | SLC6A4 | ESCITALOPRAM | inhibitor |
| SLC6A4 | SLC6A4 | SLC6A4 | BUPROPION |  |
| SLC6A4 | SLC6A4 | SLC6A4 | CLOZAPINE |  |
| SLC6A4 | SLC6A4 | SLC6A4 | PROTRIPTYLINE | inhibitor |
| SLC6A4 | SLC6A4 | SLC6A4 | TRIMIPRAMINE | inhibitor |
| SLC6A4 | SLC6A4 | SLC6A4 | ONDANSETRON |  |
| SLC6A4 | SLC6A4 | SLC6A4 | PSEUDOEPHEDRINE | inhibitor |
| SLC6A4 | SLC6A4 | SLC6A4 | VORTIOXETINE | inhibitor |
| SLC6A4 | SLC6A4 | SLC6A4 | RIBAVIRIN |  |
| SLC6A4 | SLC6A4 | SLC6A4 | HALOPERIDOL |  |
| SLC6A4 | SLC6A4 | SLC6A4 | IMIPRAMINE | inhibitor |
| SLC6A4 | SLC6A4 | SLC6A4 | PAROXETINE | inhibitor |
| SLC6A4 | SLC6A4 | SLC6A4 | SOLRIAMFETOL |  |
| SLC6A4 | SLC6A4 | SLC6A4 | FLUVOXAMINE | inhibitor |
| SLC6A4 | SLC6A4 | SLC6A4 | VENLAFAXINE | inhibitor |
| SLC6A4 | SLC6A4 | SLC6A4 | NORTRIPTYLINE | inhibitor |
| SLC6A4 | SLC6A4 | SLC6A4 | BUPRENORPHINE |  |
| SLC6A4 | SLC6A4 | SLC6A4 | OLANZAPINE |  |
| SLC6A4 | SLC6A4 | SLC6A4 | DESIPRAMINE | inhibitor |
| SLC6A4 | SLC6A4 | SLC6A4 | COCAINE | inhibitor |
| SLC6A4 | SLC6A4 | SLC6A4 | METHADONE |  |
| SLC6A4 | SLC6A4 | SLC6A4 | ALCOHOL |  |
| SLC6A4 | SLC6A4 | SLC6A4 | PHENTERMINE | inhibitor |
| SLC6A4 | SLC6A4 | SLC6A4 | TRAMADOL | inhibitor |
| HTR6 | HTR6 | HTR6 | RISPERIDONE | antagonist |
| HTR6 | HTR6 | HTR6 | ILOPERIDONE | antagonist |
| HTR6 | HTR6 | HTR6 | ASENAPINE | antagonist |
| HTR6 | HTR6 | HTR6 | OLANZAPINE | antagonist |
| CACNA2D3 | CACNA2D3 | CACNA2D3 | PREGABALIN | modulator |
| CACNA2D3 | CACNA2D3 | CACNA2D3 | BEPRIDIL HYDROCHLORIDE | blocker |
| CACNA2D3 | CACNA2D3 | CACNA2D3 | GABAPENTIN | modulator |
| CACNA2D3 | CACNA2D3 | CACNA2D3 | GABAPENTIN ENACARBIL | modulator |
| PIN1 | PIN1 | PIN1 | IRINOTECAN |  |
| PIN1 | PIN1 | PIN1 | OXALIPLATIN |  |
| IL3RA | IL3RA | IL3RA | TAGRAXOFUSP | binder |
| IL3RA | IL3RA | IL3RA | EPOETIN ALFA |  |
| NR1H3 | NR1H3 | NR1H3 | ATENOLOL |  |
| NR1H3 | NR1H3 | NR1H3 | BEXAROTENE |  |
| NR1H3 | NR1H3 | NR1H3 | VERAPAMIL |  |
| IRAK1 | IRAK1 | IRAK1 | SORAFENIB |  |
| IRAK1 | IRAK1 | IRAK1 | GEFITINIB |  |
| IRAK1 | IRAK1 | IRAK1 | IMATINIB |  |
| CARTPT | CARTPT | CARTPT | INSULIN |  |
| CARTPT | CARTPT | CARTPT | AMPHETAMINE | agonist |
| CARTPT | CARTPT | CARTPT | DEXAMETHASONE |  |
| CARTPT | CARTPT | CARTPT | PROGESTERONE |  |
| IL1A | IL1A | IL1A | OLANZAPINE |  |
| IL1A | IL1A | IL1A | RILONACEPT | binder |
| IL1A | IL1A | IL1A | HYDROXYCHLOROQUINE |  |
| NOD2 | NOD2 | NOD2 | TACROLIMUS |  |
| NOD2 | NOD2 | NOD2 | MIFAMURTIDE | ligand |
| PSMC6 | PSMC6 | PSMC6 | CARFILZOMIB | inhibitor |
| PSMC6 | PSMC6 | PSMC6 | IXAZOMIB CITRATE | inhibitor |
| PSMC6 | PSMC6 | PSMC6 | BORTEZOMIB | inhibitor |
| B4GALT2 | B4GALT2 | B4GALT2 | ASPIRIN |  |
| B4GALT2 | B4GALT2 | B4GALT2 | CLOPIDOGREL |  |
| BRD2 | BRD2 | BRD2 | ACETAMINOPHEN |  |
| BRD2 | BRD2 | BRD2 | ALPRAZOLAM |  |
| BRD2 | BRD2 | BRD2 | MIDAZOLAM |  |
| SIRT1 | SIRT1 | SIRT1 | NIACINAMIDE |  |
| CDK5 | CDK5 | CDK5 | PALBOCICLIB |  |
| FDFT1 | FDFT1 | FDFT1 | LOVASTATIN |  |
| KLK1 | KLK1 | KLK1 | ECALLANTIDE |  |
| VWF | VWF | VWF | RIBAVIRIN |  |
| VWF | VWF | VWF | ACETYLCYSTEINE |  |
| VWF | VWF | VWF | MITOMYCIN |  |
| VWF | VWF | VWF | PREDNISONE |  |
| VWF | VWF | VWF | STREPTOZOCIN |  |
| VWF | VWF | VWF | THALIDOMIDE |  |
| VWF | VWF | VWF | WARFARIN |  |
| VWF | VWF | VWF | PENTOXIFYLLINE |  |
| VWF | VWF | VWF | CAPLACIZUMAB | inhibitor |
| VWF | VWF | VWF | PHENYLEPHRINE |  |
| VWF | VWF | VWF | VINCRISTINE |  |
| PSMC5 | PSMC5 | PSMC5 | CARFILZOMIB | inhibitor |
| PSMC5 | PSMC5 | PSMC5 | BORTEZOMIB | inhibitor |
| PSMC5 | PSMC5 | PSMC5 | IXAZOMIB | inhibitor |
| PSMC5 | PSMC5 | PSMC5 | IXAZOMIB CITRATE | inhibitor |
| GANAB | GANAB | GANAB | MIGLUSTAT |  |
| NDUFV2 | NDUFV2 | NDUFV2 | METFORMIN HYDROCHLORIDE | inhibitor |
| MICB | MICB | MICB | RIBAVIRIN |  |
| RPS6KA3 | RPS6KA3 | RPS6KA3 | UREA |  |
| RPS6KA3 | RPS6KA3 | RPS6KA3 | PALBOCICLIB |  |
| SDF4 | SDF4 | SDF4 | ALCOHOL |  |
| CD4 | CD4 | CD4 | IBALIZUMAB | antagonist|inhibitor|antibody |
| CXCR2 | CXCR2 | CXCR2 | CLOTRIMAZOLE |  |
| CXCR2 | CXCR2 | CXCR2 | ACETYLCYSTEINE |  |
| CXCR2 | CXCR2 | CXCR2 | IBUPROFEN |  |
| MAP3K5 | MAP3K5 | MAP3K5 | HYDROXYUREA |  |
| BAG6 | BAG6 | BAG6 | CARBAMAZEPINE |  |
| HBEGF | HBEGF | HBEGF | CETUXIMAB |  |
| HBEGF | HBEGF | HBEGF | PANITUMUMAB |  |
| HSD17B10 | HSD17B10 | HSD17B10 | EPINEPHRINE BITARTRATE |  |
| HSD17B10 | HSD17B10 | HSD17B10 | SULFAPHENAZOLE |  |
| HSD17B10 | HSD17B10 | HSD17B10 | PSEUDOEPHEDRINE |  |
| HSD17B10 | HSD17B10 | HSD17B10 | TELMISARTAN |  |
| HSD17B10 | HSD17B10 | HSD17B10 | METHOTREXATE |  |
| HSD17B10 | HSD17B10 | HSD17B10 | CARBARIL |  |
| HSD17B10 | HSD17B10 | HSD17B10 | THIMEROSAL |  |
| HSD17B10 | HSD17B10 | HSD17B10 | HYDROCORTISONE |  |
| HSD17B10 | HSD17B10 | HSD17B10 | BUMETANIDE |  |
| HSD17B10 | HSD17B10 | HSD17B10 | AMSACRINE |  |
| HSD17B10 | HSD17B10 | HSD17B10 | LEVODOPA |  |
| HSD17B10 | HSD17B10 | HSD17B10 | HALOTHANE |  |
| HSD17B10 | HSD17B10 | HSD17B10 | RALOXIFENE |  |
| HSD17B10 | HSD17B10 | HSD17B10 | PROTRIPTYLINE |  |
| HSD17B10 | HSD17B10 | HSD17B10 | DOPAMINE |  |
| HSD17B10 | HSD17B10 | HSD17B10 | PREDNISONE |  |
| HSD17B10 | HSD17B10 | HSD17B10 | CIPROFLOXACIN |  |
| HSD17B10 | HSD17B10 | HSD17B10 | MALATHION |  |
| HSD17B10 | HSD17B10 | HSD17B10 | METHYLDOPA |  |
| HSD17B10 | HSD17B10 | HSD17B10 | NOREPINEPHRINE BITARTRATE |  |
| HSD17B10 | HSD17B10 | HSD17B10 | METOLAZONE |  |
| HSD17B10 | HSD17B10 | HSD17B10 | HYDROXYZINE PAMOATE |  |
| HSD17B10 | HSD17B10 | HSD17B10 | AMPHOTERICIN B |  |
| HSD17B10 | HSD17B10 | HSD17B10 | PRAZOSIN |  |
| HSD17B10 | HSD17B10 | HSD17B10 | HEXACHLOROPHENE |  |
| HSD17B10 | HSD17B10 | HSD17B10 | OXYTETRACYCLINE HYDROCHLORIDE |  |
| HSD17B10 | HSD17B10 | HSD17B10 | TRAZODONE |  |
| HSD17B10 | HSD17B10 | HSD17B10 | RIBOFLAVIN |  |
| HSD17B10 | HSD17B10 | HSD17B10 | MYCOPHENOLIC ACID |  |
| HSD17B10 | HSD17B10 | HSD17B10 | BENZBROMARONE |  |
| HSD17B10 | HSD17B10 | HSD17B10 | CISPLATIN |  |
| HSD17B10 | HSD17B10 | HSD17B10 | LABETALOL HYDROCHLORIDE |  |
| HSD17B10 | HSD17B10 | HSD17B10 | GRISEOFULVIN |  |
| HSD17B10 | HSD17B10 | HSD17B10 | LEVONORDEFRIN |  |
| HSD17B10 | HSD17B10 | HSD17B10 | ZOLPIDEM |  |
| HSD17B10 | HSD17B10 | HSD17B10 | FOLIC ACID |  |
| HSD17B10 | HSD17B10 | HSD17B10 | DIFLUNISAL |  |
| HSD17B10 | HSD17B10 | HSD17B10 | RALOXIFENE HYDROCHLORIDE |  |
| HSD17B10 | HSD17B10 | HSD17B10 | INAMRINONE |  |
| HSD17B10 | HSD17B10 | HSD17B10 | CARBOPLATIN |  |
| HSD17B10 | HSD17B10 | HSD17B10 | ALFUZOSIN |  |
| HSD17B10 | HSD17B10 | HSD17B10 | OFLOXACIN |  |
| HSD17B10 | HSD17B10 | HSD17B10 | ERGONOVINE |  |
| HSD17B10 | HSD17B10 | HSD17B10 | MESALAMINE |  |
| HSD17B10 | HSD17B10 | HSD17B10 | DEQUALINIUM |  |
| HSD17B10 | HSD17B10 | HSD17B10 | PRAZOSIN HYDROCHLORIDE |  |
| HSD17B10 | HSD17B10 | HSD17B10 | NIFEDIPINE |  |
| HSD17B10 | HSD17B10 | HSD17B10 | EPINEPHRINE |  |
| HSD17B10 | HSD17B10 | HSD17B10 | AMILORIDE HYDROCHLORIDE |  |
| HSD17B10 | HSD17B10 | HSD17B10 | AMILORIDE |  |
| HSD17B10 | HSD17B10 | HSD17B10 | PIRFENIDONE |  |
| HSD17B10 | HSD17B10 | HSD17B10 | CARBIDOPA |  |
| HSD17B10 | HSD17B10 | HSD17B10 | PYROGALLOL |  |
| HSD17B10 | HSD17B10 | HSD17B10 | DISULFIRAM |  |
| HSD17B10 | HSD17B10 | HSD17B10 | GLIQUIDONE |  |
| HSD17B10 | HSD17B10 | HSD17B10 | PIRETANIDE |  |
| HSD17B10 | HSD17B10 | HSD17B10 | SALMETEROL XINAFOATE |  |
| HSD17B10 | HSD17B10 | HSD17B10 | TRAZODONE HYDROCHLORIDE |  |
| HSD17B10 | HSD17B10 | HSD17B10 | ACRISORCIN |  |
| HSD17B10 | HSD17B10 | HSD17B10 | TRIAMTERENE |  |
| HSD17B10 | HSD17B10 | HSD17B10 | ETHAMSYLATE |  |
| HSD17B10 | HSD17B10 | HSD17B10 | HYDROQUINONE |  |
| HSD17B10 | HSD17B10 | HSD17B10 | APOMORPHINE |  |
| HSD17B10 | HSD17B10 | HSD17B10 | PADIMATE O |  |
| HSD17B10 | HSD17B10 | HSD17B10 | DIPYRIDAMOLE |  |
| PSMA7 | PSMA7 | PSMA7 | CARFILZOMIB | inhibitor |
| PSMA7 | PSMA7 | PSMA7 | IXAZOMIB CITRATE | inhibitor |
| PSMA7 | PSMA7 | PSMA7 | BORTEZOMIB | inhibitor |
| PSMD4 | PSMD4 | PSMD4 | CARFILZOMIB | inhibitor |
| PSMD4 | PSMD4 | PSMD4 | IXAZOMIB CITRATE | inhibitor |
| PSMD4 | PSMD4 | PSMD4 | BORTEZOMIB | inhibitor |
| PSMD4 | PSMD4 | PSMD4 | TALAZOPARIB |  |
| NDUFA3 | NDUFA3 | NDUFA3 | METFORMIN HYDROCHLORIDE | inhibitor |
| PTAFR | PTAFR | PTAFR | TICLOPIDINE |  |
| PIK3CD | PIK3CD | PIK3CD | COPANLISIB | inhibitor |
| PIK3CD | PIK3CD | PIK3CD | DUVELISIB | inhibitor |
| PIK3CD | PIK3CD | PIK3CD | ALPELISIB | inhibitor |
| PIK3CD | PIK3CD | PIK3CD | IDELALISIB | inhibitor |
| HLA-DQB1 | HLA-DQB1 | HLA-DQB1 | TICLOPIDINE |  |
| HLA-DQB1 | HLA-DQB1 | HLA-DQB1 | CLAVULANIC ACID |  |
| HLA-DQB1 | HLA-DQB1 | HLA-DQB1 | NEVIRAPINE |  |
| HLA-DQB1 | HLA-DQB1 | HLA-DQB1 | AMOXICILLIN |  |
| HLA-DQB1 | HLA-DQB1 | HLA-DQB1 | LAMOTRIGINE |  |
| HLA-DQB1 | HLA-DQB1 | HLA-DQB1 | ASPIRIN |  |
| HLA-DQB1 | HLA-DQB1 | HLA-DQB1 | FLOXACILLIN |  |
| HLA-DQB1 | HLA-DQB1 | HLA-DQB1 | CARBAMAZEPINE |  |
| HLA-DQB1 | HLA-DQB1 | HLA-DQB1 | ACETAMINOPHEN |  |
| NDUFB8 | NDUFB8 | NDUFB8 | METFORMIN HYDROCHLORIDE | inhibitor |
| IL2RG | IL2RG | IL2RG | DACLIZUMAB | inhibitor |
| IL2RG | IL2RG | IL2RG | ALDESLEUKIN | agonist |
| IL2RG | IL2RG | IL2RG | BASILIXIMAB | inhibitor |
| IL2RG | IL2RG | IL2RG | DENILEUKIN DIFTITOX |  |
| CRBN | CRBN | CRBN | POMALIDOMIDE | inhibitor |
| CRBN | CRBN | CRBN | LENALIDOMIDE | inhibitor |
| CRBN | CRBN | CRBN | THALIDOMIDE | inhibitor |
| XBP1 | XBP1 | XBP1 | FLUSPIRILENE |  |
| XBP1 | XBP1 | XBP1 | PIMOZIDE |  |
| IL6R | IL6R | IL6R | BAZEDOXIFENE |  |
| IL6R | IL6R | IL6R | RALOXIFENE |  |
| IL6R | IL6R | IL6R | TOCILIZUMAB | antibody|inhibitor |
| IL6R | IL6R | IL6R | SARILUMAB | antagonist|antibody |
| IL6R | IL6R | IL6R | THALIDOMIDE |  |
| IL6R | IL6R | IL6R | FLUOROURACIL |  |
| NDUFA13 | NDUFA13 | NDUFA13 | METFORMIN HYDROCHLORIDE | inhibitor |
| MDH2 | MDH2 | MDH2 | CISPLATIN |  |
| MDH2 | MDH2 | MDH2 | ALBUMIN HUMAN |  |
| FCGR3B | FCGR3B | FCGR3B | PROGESTERONE |  |
| FCGR3B | FCGR3B | FCGR3B | SODIUM CHLORIDE |  |
| FCGR3B | FCGR3B | FCGR3B | CYCLOSPORINE |  |
| FCGR3B | FCGR3B | FCGR3B | INDOMETHACIN |  |
| FCGR3B | FCGR3B | FCGR3B | METHOTREXATE |  |
| FCGR3B | FCGR3B | FCGR3B | PREDNISOLONE |  |
| FCGR3B | FCGR3B | FCGR3B | THALIDOMIDE |  |
| FCGR3B | FCGR3B | FCGR3B | CHOLECALCIFEROL |  |
| FCGR3B | FCGR3B | FCGR3B | EPOETIN ALFA |  |
| FCGR3B | FCGR3B | FCGR3B | PENICILLIN G POTASSIUM |  |
| FCGR3B | FCGR3B | FCGR3B | METHIMAZOLE |  |
| FCGR3B | FCGR3B | FCGR3B | LACTULOSE |  |
| FCGR3B | FCGR3B | FCGR3B | FENTANYL |  |
| FCGR3B | FCGR3B | FCGR3B | DOXORUBICIN |  |
| SYK | SYK | SYK | FOSTAMATINIB | inhibitor |
| SYK | SYK | SYK | IMATINIB MESYLATE |  |
| SYK | SYK | SYK | DASATINIB |  |
| SYK | SYK | SYK | ERLOTINIB |  |
| SYK | SYK | SYK | PACLITAXEL |  |
| UBE2N | UBE2N | UBE2N | PERPHENAZINE |  |
| UBE2N | UBE2N | UBE2N | RALOXIFENE HYDROCHLORIDE |  |
| UBE2N | UBE2N | UBE2N | THONZONIUM BROMIDE |  |
| ARF6 | ARF6 | ARF6 | INSULIN |  |
| ARID1A | ARID1A | ARID1A | NIVOLUMAB |  |
| ARID1A | ARID1A | ARID1A | PEMBROLIZUMAB |  |
| ARID1A | ARID1A | ARID1A | ATEZOLIZUMAB |  |
| ARID1A | ARID1A | ARID1A | SORAFENIB |  |
| ARID1A | ARID1A | ARID1A | DASATINIB |  |
| CD55 | CD55 | CD55 | ALCOHOL |  |
| YWHAE | YWHAE | YWHAE | INSULIN |  |
| PRKCD | PRKCD | PRKCD | ALCOHOL |  |
| PRKCD | PRKCD | PRKCD | MIDOSTAURIN | inhibitor |
| CTDSP1 | CTDSP1 | CTDSP1 | ISOPROPAMIDE IODIDE |  |
| CTDSP1 | CTDSP1 | CTDSP1 | CEFUROXIME SODIUM |  |
| CTDSP1 | CTDSP1 | CTDSP1 | CEPHALOTHIN |  |
| CTDSP1 | CTDSP1 | CTDSP1 | CEFACLOR |  |
| CTDSP1 | CTDSP1 | CTDSP1 | CEFDINIR |  |
| RBL2 | RBL2 | RBL2 | SIROLIMUS |  |
| PSMB4 | PSMB4 | PSMB4 | CARFILZOMIB | inhibitor |
| PSMB4 | PSMB4 | PSMB4 | BORTEZOMIB | inhibitor |
| PSMB4 | PSMB4 | PSMB4 | IXAZOMIB CITRATE | inhibitor |
| WRB | GET1 | GET1 | METHYLPHENIDATE |  |
| STAT6 | STAT6 | STAT6 | DOXORUBICIN HYDROCHLORIDE |  |
| STAT6 | STAT6 | STAT6 | INDOMETHACIN |  |
| PSMA2 | PSMA2 | PSMA2 | BORTEZOMIB | inhibitor |
| PSMA2 | PSMA2 | PSMA2 | CARFILZOMIB | inhibitor |
| PSMA2 | PSMA2 | PSMA2 | IXAZOMIB CITRATE | inhibitor |
| CAST | CAST | CAST | THROMBIN |  |
| CAST | CAST | CAST | EPINEPHRINE |  |
| CAPG | CAPG | CAPG | VINCRISTINE |  |
| RXRA | RXRA | RXRA | TRICLOSAN |  |
| RXRA | RXRA | RXRA | RALOXIFENE HYDROCHLORIDE |  |
| RXRA | RXRA | RXRA | DOXORUBICIN HYDROCHLORIDE |  |
| RXRA | RXRA | RXRA | DOCETAXEL |  |
| RXRA | RXRA | RXRA | PHENOXYETHANOL |  |
| RXRA | RXRA | RXRA | PROGESTERONE |  |
| RXRA | RXRA | RXRA | ACITRETIN | agonist |
| RXRA | RXRA | RXRA | IMIPRAMINE HYDROCHLORIDE |  |
| RXRA | RXRA | RXRA | THIMEROSAL |  |
| RXRA | RXRA | RXRA | TRETINOIN |  |
| RXRA | RXRA | RXRA | DAUNORUBICIN HYDROCHLORIDE |  |
| RXRA | RXRA | RXRA | DACTINOMYCIN |  |
| RXRA | RXRA | RXRA | ALITRETINOIN | agonist |
| RXRA | RXRA | RXRA | TRICLOCARBAN |  |
| RXRA | RXRA | RXRA | ADAPALENE | agonist |
| RXRA | RXRA | RXRA | NICLOSAMIDE |  |
| RXRA | RXRA | RXRA | SULINDAC | antagonist |
| RXRA | RXRA | RXRA | BEXAROTENE | activator|agonist |
| RXRA | RXRA | RXRA | PIMOZIDE |  |
| RXRA | RXRA | RXRA | SAQUINAVIR MESYLATE |  |
| RXRA | RXRA | RXRA | TESTOSTERONE |  |
| HDAC1 | HDAC1 | HDAC1 | PANOBINOSTAT | inhibitor |
| HDAC1 | HDAC1 | HDAC1 | DAUNORUBICIN |  |
| HDAC1 | HDAC1 | HDAC1 | ROMIDEPSIN | antagonist|inhibitor |
| HDAC1 | HDAC1 | HDAC1 | VORINOSTAT | inhibitor |
| HDAC1 | HDAC1 | HDAC1 | BELINOSTAT | inhibitor |
| HDAC1 | HDAC1 | HDAC1 | PANOBINOSTAT LACTATE | inhibitor |
| HDAC1 | HDAC1 | HDAC1 | VALPROIC ACID |  |
| HDAC1 | HDAC1 | HDAC1 | PHENYLBUTANOIC ACID |  |
| SUMO4 | SUMO4 | SUMO4 | TACROLIMUS |  |
| HSP90AB1 | HSP90AB1 | HSP90AB1 | DIPYRIDAMOLE |  |
| HSP90AB1 | HSP90AB1 | HSP90AB1 | DIACEREIN |  |
| HSP90AB1 | HSP90AB1 | HSP90AB1 | DAUNORUBICIN HYDROCHLORIDE |  |
| HSP90AB1 | HSP90AB1 | HSP90AB1 | BEVACIZUMAB |  |
| HSP90AB1 | HSP90AB1 | HSP90AB1 | DOXORUBICIN HYDROCHLORIDE |  |
| CXCR4 | CXCR4 | CXCR4 | BEVACIZUMAB |  |
| CXCR4 | CXCR4 | CXCR4 | PLERIXAFOR | partial agonist|antagonist |
| CXCR4 | CXCR4 | CXCR4 | CISPLATIN |  |
| PKM | PKM | PKM | HYDRALAZINE |  |
| PKM | PKM | PKM | SULFADIAZINE, SILVER |  |
| PKM | PKM | PKM | NICLOSAMIDE |  |
| PKM | PKM | PKM | PSEUDOEPHEDRINE |  |
| PKM | PKM | PKM | NITAZOXANIDE |  |
| PKM | PKM | PKM | AMLEXANOX |  |
| CDC42 | CDC42 | CDC42 | GONADORELIN ACETATE |  |
| RAB27A | RAB27A | RAB27A | EMAPALUMAB |  |
| FCER1A | FCER1A | FCER1A | OMALIZUMAB | inhibitor |
| FCER1A | FCER1A | FCER1A | DESLORATADINE |  |
| FCER1A | FCER1A | FCER1A | MIZOLASTINE |  |
| TOP2B | TOP2B | TOP2B | ETOPOSIDE | inhibitor |
| TOP2B | TOP2B | TOP2B | ROSOXACIN |  |
| TOP2B | TOP2B | TOP2B | DEXRAZOXANE |  |
| TOP2B | TOP2B | TOP2B | MITOXANTRONE |  |
| TOP2B | TOP2B | TOP2B | VALRUBICIN |  |
| TOP2B | TOP2B | TOP2B | CINOXACIN |  |
| TOP2B | TOP2B | TOP2B | DIGITOXIN |  |
| TOP2B | TOP2B | TOP2B | LOMEFLOXACIN |  |
| TOP2B | TOP2B | TOP2B | DOXORUBICIN |  |
| TOP2B | TOP2B | TOP2B | AMSACRINE |  |
| TOP2B | TOP2B | TOP2B | ENOXACIN |  |
| TOP2B | TOP2B | TOP2B | IDARUBICIN |  |
| TOP2B | TOP2B | TOP2B | PEFLOXACIN |  |
| TOP2B | TOP2B | TOP2B | EPIRUBICIN |  |
| TOP2B | TOP2B | TOP2B | PODOFILOX |  |
| TOP2B | TOP2B | TOP2B | TENIPOSIDE | inhibitor |
| TOP2B | TOP2B | TOP2B | DAUNORUBICIN | inhibitor |
| TOP2B | TOP2B | TOP2B | PIXANTRONE |  |
| P4HB | P4HB | P4HB | RALOXIFENE HYDROCHLORIDE |  |
| P4HB | P4HB | P4HB | LOMITAPIDE MESYLATE | inhibitor |
| GZMB | GZMB | GZMB | HEXACHLOROPHENE |  |
| FKBP5 | FKBP5 | FKBP5 | NEFAZODONE |  |
| FKBP5 | FKBP5 | FKBP5 | CLOZAPINE |  |
| FKBP5 | FKBP5 | FKBP5 | VENLAFAXINE |  |
| FKBP5 | FKBP5 | FKBP5 | PAROXETINE |  |
| FKBP5 | FKBP5 | FKBP5 | CITALOPRAM |  |
| FKBP5 | FKBP5 | FKBP5 | BUPROPION |  |
| FKBP5 | FKBP5 | FKBP5 | CLOMIPRAMINE |  |
| FKBP5 | FKBP5 | FKBP5 | FLUOXETINE |  |
| FKBP5 | FKBP5 | FKBP5 | GEMCITABINE |  |
| FKBP5 | FKBP5 | FKBP5 | ESCITALOPRAM |  |
| FKBP5 | FKBP5 | FKBP5 | MIRTAZAPINE |  |
| HINT1 | HINT1 | HINT1 | NICOTINE |  |
| CSK | CSK | CSK | HYDROCHLOROTHIAZIDE |  |
| ITGAL | ITGAL | ITGAL | FLUOROURACIL |  |
| ITGAL | ITGAL | ITGAL | EPOETIN ALFA |  |
| ITGAL | ITGAL | ITGAL | ETOPOSIDE |  |
| ITGAL | ITGAL | ITGAL | THROMBIN |  |
| ITGAL | ITGAL | ITGAL | MYCOPHENOLATE MOFETIL |  |
| ITGAL | ITGAL | ITGAL | CYCLOSPORINE |  |
| ITGAL | ITGAL | ITGAL | SIROLIMUS |  |
| ITGAL | ITGAL | ITGAL | CYCLOPHOSPHAMIDE |  |
| ITGAL | ITGAL | ITGAL | BUSULFAN |  |
| ITGAL | ITGAL | ITGAL | LIFITEGRAST | antagonist |
| VASP | VASP | VASP | HYDROCHLOROTHIAZIDE |  |
| IL17RA | IL17RA | IL17RA | BRODALUMAB | antagonist|antibody |
| RAC1 | RAC1 | RAC1 | DABRAFENIB |  |
| RAC1 | RAC1 | RAC1 | VEMURAFENIB |  |
| CCR2 | CCR2 | CCR2 | MORPHINE |  |
| CCR2 | CCR2 | CCR2 | PLERIXAFOR |  |
| CCR2 | CCR2 | CCR2 | SIMVASTATIN |  |
| FYN | FYN | FYN | NINTEDANIB | inhibitor |
| FYN | FYN | FYN | VANDETANIB |  |
| FYN | FYN | FYN | DASATINIB | inhibitor|multitarget |
| FYN | FYN | FYN | PAZOPANIB |  |
| NDUFA1 | NDUFA1 | NDUFA1 | METFORMIN HYDROCHLORIDE | inhibitor |
| ARRB2 | ARRB2 | ARRB2 | FENTANYL |  |
| ARRB2 | ARRB2 | ARRB2 | BUPRENORPHINE |  |
| ARRB2 | ARRB2 | ARRB2 | TRAMADOL |  |
| ICAM3 | ICAM3 | ICAM3 | CYCLOSPORINE |  |
| ICAM3 | ICAM3 | ICAM3 | METHOTREXATE |  |
| GSTP1 | GSTP1 | GSTP1 | ALCOHOL |  |
| GSTP1 | GSTP1 | GSTP1 | DAUNORUBICIN |  |
| GSTP1 | GSTP1 | GSTP1 | LEUCOVORIN |  |
| GSTP1 | GSTP1 | GSTP1 | CYTARABINE |  |
| GSTP1 | GSTP1 | GSTP1 | ISONIAZID |  |
| GSTP1 | GSTP1 | GSTP1 | BUSULFAN |  |
| GSTP1 | GSTP1 | GSTP1 | PREDNISONE |  |
| GSTP1 | GSTP1 | GSTP1 | MELPHALAN |  |
| GSTP1 | GSTP1 | GSTP1 | VERAPAMIL |  |
| GSTP1 | GSTP1 | GSTP1 | THIOTEPA |  |
| GSTP1 | GSTP1 | GSTP1 | DECITABINE |  |
| GSTP1 | GSTP1 | GSTP1 | IFOSFAMIDE |  |
| GSTP1 | GSTP1 | GSTP1 | CISPLATIN |  |
| GSTP1 | GSTP1 | GSTP1 | DEXAMETHASONE |  |
| GSTP1 | GSTP1 | GSTP1 | AZACITIDINE |  |
| GSTP1 | GSTP1 | GSTP1 | FLUOROURACIL |  |
| GSTP1 | GSTP1 | GSTP1 | RIFAMPIN |  |
| GSTP1 | GSTP1 | GSTP1 | EPIRUBICIN |  |
| GSTP1 | GSTP1 | GSTP1 | OMEPRAZOLE |  |
| GSTP1 | GSTP1 | GSTP1 | OXALIPLATIN |  |
| GSTP1 | GSTP1 | GSTP1 | CARBOPLATIN |  |
| GSTP1 | GSTP1 | GSTP1 | DOXORUBICIN |  |
| GSTP1 | GSTP1 | GSTP1 | DOCETAXEL |  |
| GSTP1 | GSTP1 | GSTP1 | PYRIMETHAMINE |  |
| GSTP1 | GSTP1 | GSTP1 | SELENOMETHIONINE |  |
| GSTP1 | GSTP1 | GSTP1 | IRINOTECAN HYDROCHLORIDE |  |
| GSTP1 | GSTP1 | GSTP1 | HYDROQUINONE |  |
| GSTP1 | GSTP1 | GSTP1 | BLEOMYCIN |  |
| GSTP1 | GSTP1 | GSTP1 | CYCLOPHOSPHAMIDE |  |
| GSTP1 | GSTP1 | GSTP1 | PACLITAXEL |  |
| GSTP1 | GSTP1 | GSTP1 | ETOPOSIDE |  |
| PGK1 | PGK1 | PGK1 | LAMIVUDINE |  |
| PPA1 | PPA1 | PPA1 | ALCOHOL |  |
| CSF3R | CSF3R | CSF3R | PEXIDARTINIB |  |
| CSF3R | CSF3R | CSF3R | LIPEGFILGRASTIM | agonist |
| CSF3R | CSF3R | CSF3R | RUXOLITINIB |  |
| CSF3R | CSF3R | CSF3R | FILGRASTIM | stimulator|agonist |
| CSF3R | CSF3R | CSF3R | PEGFILGRASTIM | agonist |
| CYBA | CYBA | CYBA | SIMVASTATIN |  |
| CYBA | CYBA | CYBA | DOXORUBICIN |  |
| CYBA | CYBA | CYBA | IDARUBICIN |  |
| IFNGR1 | IFNGR1 | IFNGR1 | INTERFERON GAMMA-1B | binder|agonist |
| CST3 | CST3 | CST3 | DIGOXIN |  |
| CST3 | CST3 | CST3 | RIBAVIRIN |  |
| PTPN6 | PTPN6 | PTPN6 | TOFACITINIB |  |
| PTPN6 | PTPN6 | PTPN6 | SORAFENIB |  |
| CSF1R | CSF1R | CSF1R | DASATINIB | inhibitor |
| CSF1R | CSF1R | CSF1R | PEXIDARTINIB | inhibitor |
| CSF1R | CSF1R | CSF1R | SUNITINIB MALATE | inhibitor |
| CSF1R | CSF1R | CSF1R | PAZOPANIB | inhibitor |
| CSF1R | CSF1R | CSF1R | IMATINIB | antagonist |
| CSF1R | CSF1R | CSF1R | SUNITINIB | inhibitor |
| CSF1R | CSF1R | CSF1R | PROGESTERONE |  |
| CSF1R | CSF1R | CSF1R | PAZOPANIB HYDROCHLORIDE | inhibitor |
| CSF1R | CSF1R | CSF1R | VEMURAFENIB |  |
| CSF1R | CSF1R | CSF1R | SORAFENIB |  |
| NPM1 | NPM1 | NPM1 | CRIZOTINIB |  |
| NPM1 | NPM1 | NPM1 | MIDOSTAURIN |  |
| NPM1 | NPM1 | NPM1 | VENETOCLAX |  |
| NPM1 | NPM1 | NPM1 | ALECTINIB |  |
| NPM1 | NPM1 | NPM1 | CERITINIB |  |
| NPM1 | NPM1 | NPM1 | TRETINOIN |  |
| NPM1 | NPM1 | NPM1 | VORINOSTAT |  |
| NPM1 | NPM1 | NPM1 | IXAZOMIB |  |
| NPM1 | NPM1 | NPM1 | LORLATINIB |  |
| CLC | CLC | CLC | MOXIDECTIN |  |
| DPYSL2 | DPYSL2 | DPYSL2 | ERLOSAMIDE |  |
| LST1 | LST1 | LST1 | ABACAVIR |  |
| ITGAM | ITGAM | ITGAM | HYDROCORTISONE |  |
| ITGAM | ITGAM | ITGAM | CLARITHROMYCIN |  |
| ITGAM | ITGAM | ITGAM | PHENYLEPHRINE |  |
| ITGAM | ITGAM | ITGAM | ATORVASTATIN |  |
| ITGAM | ITGAM | ITGAM | THEOPHYLLINE |  |
| ITGAM | ITGAM | ITGAM | DIMETHYL SULFOXIDE |  |
| ITGAM | ITGAM | ITGAM | MORPHINE |  |
| ITGAM | ITGAM | ITGAM | FENTANYL |  |
| UCP2 | UCP2 | UCP2 | SOYBEAN OIL |  |
| UCP2 | UCP2 | UCP2 | LIOTHYRONINE SODIUM |  |
| UCP2 | UCP2 | UCP2 | SODIUM CHLORIDE |  |
| CSF2RB | CSF2RB | CSF2RB | TAGRAXOFUSP |  |
| CSF2RB | CSF2RB | CSF2RB | SARGRAMOSTIM | agonist |
| RAC2 | RAC2 | RAC2 | DOXORUBICIN |  |
| RAC2 | RAC2 | RAC2 | IDARUBICIN |  |
| DUSP6 | DUSP6 | DUSP6 | TRAMETINIB |  |
| CD52 | CD52 | CD52 | ALEMTUZUMAB | antibody|inhibitor |
| HLA-DPB1 | HLA-DPB1 | HLA-DPB1 | ASPIRIN |  |
| HLA-DPB1 | HLA-DPB1 | HLA-DPB1 | CLOZAPINE |  |
| TUBA1B | TUBA1B | TUBA1B | VINCRISTINE SULFATE | inhibitor |
| TUBA1B | TUBA1B | TUBA1B | CABAZITAXEL | inhibitor |
| TUBA1B | TUBA1B | TUBA1B | VINORELBINE |  |
| TUBA1B | TUBA1B | TUBA1B | IXABEPILONE | inhibitor |
| TUBA1B | TUBA1B | TUBA1B | PACLITAXEL | inhibitor |
| TUBA1B | TUBA1B | TUBA1B | ERIBULIN MESYLATE | inhibitor |
| TUBA1B | TUBA1B | TUBA1B | VINCRISTINE |  |
| TUBA1B | TUBA1B | TUBA1B | BRENTUXIMAB VEDOTIN | inhibitor |
| TUBA1B | TUBA1B | TUBA1B | VORINOSTAT |  |
| TUBA1B | TUBA1B | TUBA1B | VINBLASTINE SULFATE | inhibitor |
| TUBA1B | TUBA1B | TUBA1B | COLCHICINE | inhibitor |
| TUBA1B | TUBA1B | TUBA1B | VINBLASTINE |  |
| TUBA1B | TUBA1B | TUBA1B | PODOFILOX |  |
| TUBA1B | TUBA1B | TUBA1B | TRASTUZUMAB EMTANSINE | inhibitor |
| TUBA1B | TUBA1B | TUBA1B | VINORELBINE TARTRATE | inhibitor |
| TUBA1B | TUBA1B | TUBA1B | DOCETAXEL | inhibitor |
| TUBA1B | TUBA1B | TUBA1B | VINFLUNINE | inhibitor |
| TUBA1C | TUBA1C | TUBA1C | BRENTUXIMAB VEDOTIN | inhibitor |
| TUBA1C | TUBA1C | TUBA1C | CABAZITAXEL | inhibitor |
| TUBA1C | TUBA1C | TUBA1C | COLCHICINE | inhibitor |
| TUBA1C | TUBA1C | TUBA1C | VINCRISTINE SULFATE | inhibitor |
| TUBA1C | TUBA1C | TUBA1C | TRASTUZUMAB EMTANSINE | inhibitor |
| TUBA1C | TUBA1C | TUBA1C | DOCETAXEL | inhibitor |
| TUBA1C | TUBA1C | TUBA1C | VINBLASTINE SULFATE | inhibitor |
| TUBA1C | TUBA1C | TUBA1C | VINCRISTINE |  |
| TUBA1C | TUBA1C | TUBA1C | VINBLASTINE |  |
| TUBA1C | TUBA1C | TUBA1C | VINORELBINE TARTRATE | inhibitor |
| TUBA1C | TUBA1C | TUBA1C | ERIBULIN MESYLATE | inhibitor |
| TUBA1C | TUBA1C | TUBA1C | VORINOSTAT |  |
| TUBA1C | TUBA1C | TUBA1C | IXABEPILONE | inhibitor |
| TUBA1C | TUBA1C | TUBA1C | PACLITAXEL | inhibitor |
| TUBA1C | TUBA1C | TUBA1C | PODOFILOX |  |
| TUBA1C | TUBA1C | TUBA1C | VINORELBINE |  |
| TUBA1C | TUBA1C | TUBA1C | VINFLUNINE | inhibitor |
| HLA-G | HLA-G | HLA-G | FLUOROURACIL |  |
| HLA-G | HLA-G | HLA-G | METHOTREXATE |  |
| HLA-G | HLA-G | HLA-G | CAPECITABINE |  |
| S100A12 | S100A12 | S100A12 | METHOTREXATE |  |
| EEF2 | EEF2 | EEF2 | DENILEUKIN DIFTITOX | inhibitor |
| S100A8 | S100A8 | S100A8 | METHOTREXATE |  |
| HLA-C | HLA-C | HLA-C | CLOZAPINE |  |
| HLA-C | HLA-C | HLA-C | RIBAVIRIN |  |
| HLA-C | HLA-C | HLA-C | GEMCITABINE |  |
| HLA-C | HLA-C | HLA-C | AMOXICILLIN |  |
| HLA-C | HLA-C | HLA-C | METHOTREXATE |  |
| HLA-C | HLA-C | HLA-C | LAMOTRIGINE |  |
| HLA-C | HLA-C | HLA-C | CARBOPLATIN |  |
| HLA-C | HLA-C | HLA-C | CLAVULANIC ACID |  |
| HLA-C | HLA-C | HLA-C | METHAZOLAMIDE |  |
| HLA-C | HLA-C | HLA-C | USTEKINUMAB |  |
| HLA-C | HLA-C | HLA-C | FLOXACILLIN |  |
| HLA-C | HLA-C | HLA-C | TICLOPIDINE |  |
